# I See Your Effort: Force-Related BOLD Effects in an Extended Action Execution–Observation Network Involving the Cerebellum

**DOI:** 10.1093/cercor/bhy322

**Published:** 2019-01-07

**Authors:** Letizia Casiraghi, Adnan A S Alahmadi, Anita Monteverdi, Fulvia Palesi, Gloria Castellazzi, Giovanni Savini, Karl Friston, Claudia A M Gandini Wheeler-Kingshott, Egidio D’Angelo

**Affiliations:** 1 Department of Brain and Behavioral Sciences, University of Pavia, Pavia, Italy; 2 Brain Connectivity Center, IRCCS Mondino Foundation, Pavia, Italy; 3 Diagnostic Radiography Technology Department, Faculty of Applied Medical Science, King Abdulaziz University (KAU), Jeddah 80200-21589, Saudi Arabia; 4 NMR Research Unit, Queen Square Multiple Sclerosis (MS) Centre, Department of Neuroinflammation, Institute of Neurology, University College London (UCL), London, UK; 5 Brain MRI 3T Center, Neuroradiology Unit, IRCCS Mondino Foundation, Pavia, PV, Italy; 6 Department of Electrical, Computer and Biomedical Engineering, University of Pavia, Pavia, Italy; 7 Department of Physics, University of Milan, Milan, Italy; 8 Wellcome Trust Centre for Neuroimaging, Institute of Neurology, University College London (UCL), London, UK; 9 Brain MRI 3T Mondino Research Center, IRCCS Mondino Foundation, Pavia, Italy

**Keywords:** action execution, action observation, cerebellum, functional magnetic resonance imaging, grip force

## Abstract

Action observation (AO) is crucial for motor planning, imitation learning, and social interaction, but it is not clear whether and how an action execution–observation network (AEON) processes the effort of others engaged in performing actions. In this functional magnetic resonance imaging (fMRI) study, we used a “squeeze ball” task involving different grip forces to investigate whether AEON activation showed similar patterns when executing the task or observing others performing it. Both in action execution, AE (subjects performed the visuomotor task) and action observation, AO (subjects watched a video of the task being performed by someone else), the fMRI signal was detected in cerebral and cerebellar regions. These responses showed various relationships with force mapping onto specific areas of the sensorimotor and cognitive systems. Conjunction analysis of AE and AO was repeated for the “0th” order and linear and nonlinear responses, and revealed multiple AEON nodes remapping the detection of actions, and also effort, of another person onto the observer’s own cerebrocerebellar system. This result implies that the AEON exploits the cerebellum, which is known to process sensorimotor predictions and simulations, performing an internal assessment of forces and integrating information into high-level schemes, providing a crucial substrate for action imitation.

## Introduction

Social behavior is based on understanding the actions of others and predicting appropriate reactions and subsequent interactions. In this context, perceiving the force applied to objects by others is crucial for understanding their intentions, for predicting the success of self-generated actions, and for dynamic movement control in interactions. However, there is still debate over the question of whether, when observing someone else performing an action, we mirror the actual movement dynamics or simply its goals (Calvo-Merino et al. 2006; Filimon et al. 2007; Thompson et al. 2007; Hamilton and Grafton 2008; Caspers et al. 2010; Cavallo et al. 2015; Koul et al. 2018). Understanding, through observation, the force involved in movements performed by others can prime the force imparted during subsequent action executions (AE) (Salama et al. 2011). The achievement of a better understanding of how force is represented in observation could facilitate and improve the clinical application of action observation (AO) in neurorehabilitation (Porro et al. 2007; Garrison et al. 2013; Buccino 2014). Although AE and observation have been studied using several techniques (Munzert et al. 2009; Sevdalis and Keller 2011; Vanderwert et al. 2013; Naish et al. 2014; Valchev et al. 2015), the neuronal processes involved in mirroring the motor effort of others have still not been fully explored.

The most renowned imitation learning hypothesis claims that “mirror neurons” are activated by observation of actions performed by others (Rizzolatti and Craighero 2004) and that the brain simulates the observed action by using the motor system as a forward model (Buccino et al. 2001; Miall 2003; Kilner et al. 2007; Ito 2008; Turella et al. 2009), recruiting hierarchically organized brain circuits (Kilner 2011; D’Angelo and Gandini Wheeler-Kingshott 2017). On a broader perspective, the brain has been proposed to include a “mirroring system” which can understand the intentions of others from observing movements (“body” reading) and a “mentalizing system” which can infer the intentions of others reconstructing hypothetical events (“mind” reading) (Van Overwalle and Baetens 2009; Van Overwalle, D’aes et al. 2015). In this context, even though magnetic resonance imaging (MRI) does not allow neuronal populations to be studied directly, functional MRI (fMRI), thanks to the blood-oxygenation-level-dependent (BOLD) effect (Kim and Ogawa 2012), can be used to study neuronal activation during both execution and observation of actions. fMRI studies (Buccino et al. 2001; Caspers et al. 2010; Caligiore et al. 2013; McGregor and Gribble 2015) support the notion that, during observation of a complex motor task, the AE network (AEN) and the AO network (AON) combine to form an action execution–observation network (AEON), which provides the neural infrastructure for imitation learning. Although the “core AEON” structures are the premotor cortex and a limited number of parietal and temporal cortical areas (Grèzes and Decety 2001), it is now clear that the AEON also comprises the supplementary motor area and the inferior frontal gyrus, as well as large sections of the somatosensory and occipitotemporal cortices (Gatti et al. 2017). Furthermore, the cerebellum and basal ganglia have also been suggested to play a role in an extended circuit underlying action understanding (Caligiore et al. 2013), to the point that the cerebellum is now considered to play a role as an adaptive predictor in AO (Sokolov et al. 2017). This idea derives from the general theory of cerebellar functioning, wherein the cerebellum is seen as a forward controller in behavioral schemes that concern the interaction of the body with the external world, instructing the cerebral cortex in a predictive manner (Cotterill 2001; Llinás 2009; Diedrichsen et al. 2010; Shadmehr and Mussa-Ivaldi 2012; Callan et al. 2013). Moreover, a growing body of evidence from both lesion and fMRI studies (Gazzola and Keysers 2009; Molenberghs et al. 2012; Christensen et al. 2014; Van Overwalle et al. 2014; Caligiore et al. 2017) suggests that the cerebellum plays a crucial role in action–perception coupling, coordinating the application of an appropriate force and its timing to generate movement, and thus operating in a forward mode (D’Angelo and Casali 2012; Avanzino et al. 2015; D’Angelo and Gandini Wheeler-Kingshott 2017). On these bases, it can be hypothesized that predicting how to move by observation entails processing of force and involves an extended AEON that includes the cerebellum together with a complex set of cortical areas.

In the present study, aiming to identify the network involved in force perception, we exploited a paradigm that recently showed how a complex set of linear and nonlinear BOLD responses are elicited in several brain regions, including the cerebellum, when varying the force applied to an object (Alahmadi, Pardini et al. 2015; Alahmadi, Samson et al. 2015; Alahmadi et al. 2017). We used this grip-force (GF) squeeze ball paradigm to assess whether: 1) the AON presents both linear and nonlinear BOLD-GF associations during observation of the squeeze ball task; 2) the AEN and the AON share a common neural substrate, corresponding to the extended AEON; and 3) regions identified as part of the AEON exhibit linear and nonlinear BOLD-GF associations. The results of this work indeed support the existence of force-related BOLD effects not only in AE but also in the extended AEON, which includes the cerebellum.

## Materials and Methods

### Subjects

A total of 14 right-handed healthy volunteers (9 females) were initially recruited for this study. However, 2 participants were excluded from further analysis: one who failed to follow the task instructions and another who presented head motion (translation in the *z* direction) >2 mm. The final sample thus comprised 12 subjects (7 females; mean age 26 ± 3.5 years). The handedness of each subject was evaluated using the Edinburgh handedness scaling questionnaire (Oldfield 1971); the mean laterality index was 82 (±16). All participants had normal or corrected-to-normal vision. No subject had a history of neurological or psychiatric disease. All the participants received a detailed explanation of the experimental procedures before participating in the experiment. The local research and ethics committee approved the study and all participants gave their written informed consent.

### MRI Scanner and Scanning Sequences

A 3 T Philips Achieva MR scanner (Philips Healthcare, Best, The Netherlands) with a 32-channel head coil was used to perform a 3D T1-weighted anatomical scan and 3 T2*-weighted echo-planner imaging (EPI) fMRI scans. The 3D T1-weighted sequence acquisition parameters were as follows: 3D inversion-recovery prepared gradient-echo (fast field echo) sequence with inversion time (TI) 824 ms, echo time (TE)/repetition time (TR) 3.1/6.9 ms, flip angle 8° and voxel size 1 mm isotropic. The fMRI acquisition parameters were: TR/TE 2.5/35 ms, 2.7 mm thick slices with interslice gap of 0.3 mm positioned axial-oblique to include the cerebellum, 3 × 3 mm^2^ in-plane resolution, field of view 192 × 192 mm^2^, SENSE factor 2, flip angle 90° and 200 repeated volumes.

### Experimental Design

**Figure 1.**
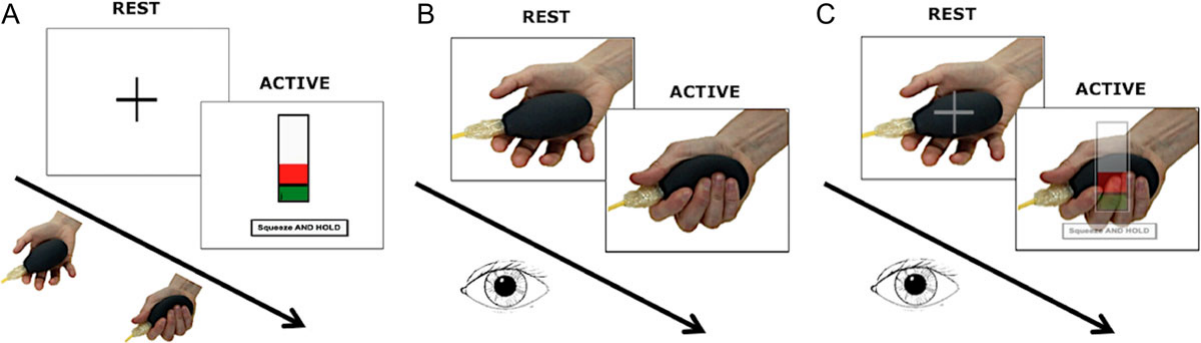
Experimental paradigm. The figure shows a pictorial representation of the 3 conditions that were used in the behavioral and fMRI sessions: (*A*) action execution (AE), (*B*) action observation (AO), and (*C*) action observation with visual cue (AOvc). The stimuli are shown above the arrow whereas the activity of the subject is shown below the arrow. During fMRI, every session lasted 8:33 min and the trials were administered in a counterbalanced and randomized order. The active trials (each lasting 3 s) were repeated 75 times and were divided equally between the 5 grip forces. A rest time of 2–12 s was allowed between active trials.

All the participants completed 3 randomized event-related fMRI sessions (Fig. 1): AE, AO, and AO with visual cue (AOvc). In all cases, all the stimuli were projected onto the same white screen, which was kept in the same position throughout; short-sighted participants used nonmagnetic visual aid goggles. The 3 experimental sessions are described below.

### Squeeze Ball Event-Related Paradigm

This paradigm, previously described elsewhere (Alahmadi, Pardini et al. 2015; Alahmadi, Samson et al. 2015; Alahmadi et al. 2017), consisted of a visuomotor event-related power grip task, in which the order and timing of trials and rest periods was optimized to introduce temporal jittering and randomization of the applied GF strength (see below). The task was performed using an MR-compatible sphygmomanometer inflation bulb (“squeeze ball”) connected to a computer suite (located outside the scanner room) running the fMRI paradigm presentation. Compression of the ball resulted in an air pressure measurement proportional to the force exerted, which was recorded at a sampling rate of 20 Hz. In all, 75 active trials were performed, divided equally into sets of 15 corresponding, respectively, to GF levels representing 20%, 30%, 40%, 50%, and 60% of the subject’s maximum voluntary contraction (MVC). MVC had previously been measured in each subject using the same force device (i.e., by asking the subject to continuously squeeze the power ball) and this value was used to set the GF target for each trial.

Trials were performed in a counterbalanced and randomized order as obtained using the OptSeq optimization software (https://surfer.nmr.mgh.harvard.edu/optseq/). The rest time between squeezing trials—this lasted between a minimum of 2 s and a maximum of 12 s, and was cued by a black crosshair located at the center of the screen—was also randomized to introduce temporal jittering between the task and data acquisition. Rest time accounted for 55% of the whole fMRI session (500 s). The visual cue used in the trials was a black static horizontal bar (presented for 3 s), which indicated the target GF level to reach. This cue was projected onto an MR-compatible white screen and shown together with an interactive colored bar, indicating the actual force level reached and thus providing real-time feedback to the subject about his/her own performance. The GF task was performed with the right (dominant) hand during the AE session.

A female actor was also filmed while performing the task in the control room of the scanner suite. The resulting video showed her whole right hand and forearm, filmed against a plain colored background, with the palm facing up. While recording the task, the computer also recorded the visual feedback she received (i.e., the visual cue bar), which was used to create a further video (in which the cue bar was superimposed on the forearm and hand) to be used in the AOvc session. Premiere Pro CS5 (Adobe System Software, CA, USA) was used for video editing.

### AO (AO and AOvc) Behavioral Sessions

Before and after the fMRI sessions, subjects underwent behavioral sessions during which they were asked to watch the AO and AOvc videos (the order of presentation of the videos was randomized among subjects) and to verbally report their own perception of the GF, that is, 20%, 30%, 40%, 50%, or 60% of the MVC of the actor’s hand shown squeezing the ball in the video (perceived GF). These sessions served to test their recognition of the GFs observed, to saturate any learning effect before the actual fMRI experiment, and to test possible differences in learning effects between the pre- and post-MRI behavioral sessions. The purpose of running 2 AO behavioral sessions, AO and AOvc, was to assess whether GFs can be appreciated from subtle (and natural) cues alone—as in the AO condition (e.g., changes in the color of the hand with increasing effort and accompanying tendon contraction)—or whether subjects also need to see symbolic visual feedback, as in the AOvc condition. Performance accuracy was assessed for each of the 5 GF levels by calculating the number of correct answers and the mean difference between the perceived GF (pGF) and the GF actually applied by the actor during the video recording (aGF).

### AE Training Session

After the AO behavioral sessions, just before the fMRI one, subjects were trained using a 2-min paradigm having a design similar to the above-described event-related one, with GF levels ranging from 10% to 70% of their MVC. The training session involved practising the task outside the scanner bore.

### AE Session

Subjects performed the AE task following the visual instructions described above. Their feedback was recorded at a sampling rate of 20 Hz during the task. The data collected served to include subject-specific performance in the statistical analysis.

### AO Session

Subjects observed the video showing the right hand of the actor performing the squeeze ball task. They were asked to keep their gaze at the center of the projection screen indicated by a cross during rest periods, to relax, not to touch the squeeze ball, and to think about nothing throughout this fMRI recording session (as opposed to trying to guess the force or the next action).

### AOvc Session

The subjects received the same instructions as in the AO session. The only difference, compared with the AO condition, concerned the stimuli: the video again showed the actor’s right hand performing the squeeze ball task, but this time the image was overlaid with a translucent representation of the visual feedback that the actor had received during the recording of the video (thus an indication of her performance). This session was originally included as part of the behavioral study as it was unclear whether force perception demands some kind of visual feedback on the performance, such as that provided by the real-time bar (symbolic guided action observation). However, since the AO condition alone was found to be sufficient to disclose perception of force-related effects, the AOvc data were not considered further for the purposes of this study.

### Data Analysis

#### Behavioral Data Analysis

The group mean accuracy of the subjects’ perceptions (pGF) was calculated overall by measuring all correct responses as a percentage of all perceived forces (at 20%, 30%, 40%, 50%, and 60% of MVC of the hand squeezing the ball in the video), and also separately for each of the following sessions: AO before MRI, AOvc before MRI, AO after MRI, and AOvc after MRI. A number of statistical tests were performed. First, we used paired sample *t*-tests to assess possible significant differences between ratings in the AO versus the AOvc sessions; that is, considering the mean accuracy of pGF in the 2 conditions (considering “AO before MRI” vs. “AOvc before MRI” and then “AO after MRI” vs. “AOvc after MRI”). Second, a repeated measures ANOVA with Bonferroni correction was implemented to investigate learning effects, that is, testing for different performances within the AO and the AOvc sessions (“AO before MRI” vs. “AO after MRI” and then “AOvc before MRI” vs. “AOvc after MRI”). A statistical threshold of *P* < 0.001 was considered significant. Finally, to characterize the challenging nature of the AO task, we assessed the correlation between the actor’s actual performance (i.e., the GF applied by the actor performing the task and recorded while filming) and the subjects’ perceptions of that GF (i.e., the pGF, as reported by each subject during the “AO before MRI” behavioral session), using the correlation coefficient (*r*) and the significance level (*P*-value). The statistical analysis was performed using the Statistical Package for the Social Sciences (SPSS) software (version 21.0).

### fMRI Data Analysis

#### Whole Brain

Image analysis was performed with SPM12 (www.fil.ion.ucl.ac.uk/spm), implemented in Matlab15b (Mathworks, Sheborn, MA), using conventional preprocessing steps: slice timing, realignment, coregistration, estimation of (nonlinear spatial) normalization parameters between the 3D T1-weighted volume and the standard SPM12 template, application of the normalization parameters to the fMRI EPI volumes, and smoothing with an 8 mm isotropic full-width half maximum (FWHM) Gaussian kernel. The GF trials were modeled as delta functions (Friston et al. 1998) with parametric modulation according to GF. A general linear model (GLM) including polynomial expansions up to the fourth order was applied following the procedures described by Alahmadi et al. (Alahmadi, Pardini et al. 2015; Alahmadi, Samson et al. 2015). As discussed in previous work, the polynomial expansion allows nonlinear relationships to be characterized in an unbiased way, by modeling a mixture of linear and nonlinear responses in a parsimonious fashion. Interpretation of the nonlinear order lends itself to hierarchical testing (e.g., second-order effects are interesting only after removing first-order effects) (Büchel et al. 1998) and neurophysiological studies have reported different response profiles that have distinct nonlinear forms (Evarts 1968; Smith et al. 1975; Cheney and Fetz 1980; Riehle et al. 1994). Moreover, polynomial expansions are the most common form of expansion (in the absence of boundary conditions) when estimating neurometric functions from imaging data (Ward and Frackowiak 2003; Kuhtz-Buschbeck et al. 2008).

In our setting, the 0th order represents the main effect of hand gripping (executed or observed) compared with the rest condition, irrespective of the level of GF applied. The first order represents any linear dependency on GF level (executed or observed), while nonlinear orders represent more complicated neurometric functions such as U-shaped (second order), sigmoid (third order) and quadratic (fourth order) functions. The parametric modulation of the stick functions—encoding grip trials—with the polynomial expansion of GF produces stimulus functions that can then be convolved with a canonical haemodynamic response function for subsequent standard GLM analyses (Friston et al. 1998).

At the first level of analysis (within subject), the realignment parameters were included in the GLM as regressors of no interest (Friston et al. 1996) and *t* statistics were used to test for the effects of each polynomial coefficient. The associated contrast images of each of the 5 polynomial coefficients were then entered into a second (between-subject) level analysis and tested with one-sample *t*-tests, following standard procedures. The same analysis pipeline was followed for the AE and AO sessions. In the AO session, the GF levels corresponded to those recorded from the actor’s performance. A voxel-wise threshold of *P_u_* < 0.001 (minimum extent 5 voxels, *P_u_*= *P* uncorrected for multiple comparisons) was used to define clusters. A threshold of *P* < 0.05 was applied to the spatial extent of clusters that survived multiple comparisons corrections. The anatomical designations of significant clusters were determined using the SPM Anatomy Toolbox (Version 2.2b). The same criteria were used for AE and AO sessions.

#### SUIT

The fMRI analysis pipeline, optimized for whole-brain analysis, can give suboptimal results in the cerebellum (Diedrichsen 2006). Therefore, to focus on the cerebellum, we used SUIT (spatially unbiased infratentorial template), a high-resolution atlas template of the human cerebellum and brainstem, which is part of the SPM12 software package (Diedrichsen et al. 2009). The following steps were performed: 1) Extraction of each subject’s cerebellum and brainstem from their corresponding whole-brain 3D T1-weighted anatomical images; 2) Normalization of the anatomical images to the SUIT template using nonlinear deformations; 3) Re-slicing of the functional contrast images produced from the first-level analysis using the deformation produced from step 2) and masking out activation outside the region of interest (i.e., the cerebellum). The normalized cerebellum functional contrast images (of each polynomial order) from each subject were then smoothed with an 8 mm FWHM Gaussian kernel and submitted to a (between-subject) standard second-level random effects analysis, testing for increasingly higher-order nonlinear effects within the cerebellum with one-sample *t*-tests. Significant clusters were defined using a height threshold of *P_u_* < 0.001 (and a minimum extent of 5 voxels). The anatomical designations of regionally specific effects were defined using a high-resolution probabilistic atlas defined within the SUIT template (Diedrichsen et al. 2009). The resulting statistical parametric maps (SPMs) were projected on to the flat map of the cerebellum provided with the SUIT template (Diedrichsen and Zotow 2015).

#### Conjunction

To identify, in terms of a parametric response to GF, the extended AEON, engaged both in AE and AO, we performed a simple conjunction analysis. This entailed testing for action observation effects at a corrected level of significance within a search volume defined by AE.

We first used the SPM, testing for 0th order effects in order to localize the combined effect of AE and AO independently of GF, that is, to identify regions that showed a conjunction of AE/AO effects, irrespective of parametric force effects. We then identified regions that showed potential nonlinear responses to GF in both action observation and execution. In order to do so, we used a full factorial design and SPMs of the *F* statistic, testing for one or more significant polynomial coefficients in both execution and observation to obtain maps of the combined AE/AO force-related effect (FRE). Specifically, we used the SPMs of the *F* statistic, testing for a parametric effect of GF under AE (threshold *P_u_*< 0.0001 for the whole brain analysis and *P_u_* < 0.001 for the SUIT analysis) as a localizing contrast to define a search region within which to identify nonlinear effects under action observation (using the equivalent *F* contrast and a small volume correction to *P* < 0.05). Finally, we used the *F* statistic of the first-order effects to investigate the linear FRE and the *F* statistic of the higher-order effects to investigate the nonlinear FRE.

## Results

BOLD fMRI signals were recorded from 12 healthy subjects during one visuomotor and 2 visual tasks for the purpose of comparing brain activation under AE and AO conditions, when different GF levels are applied to an object (in this case a squeeze ball).

### AO Behavioral Responses

**Figure 2.**
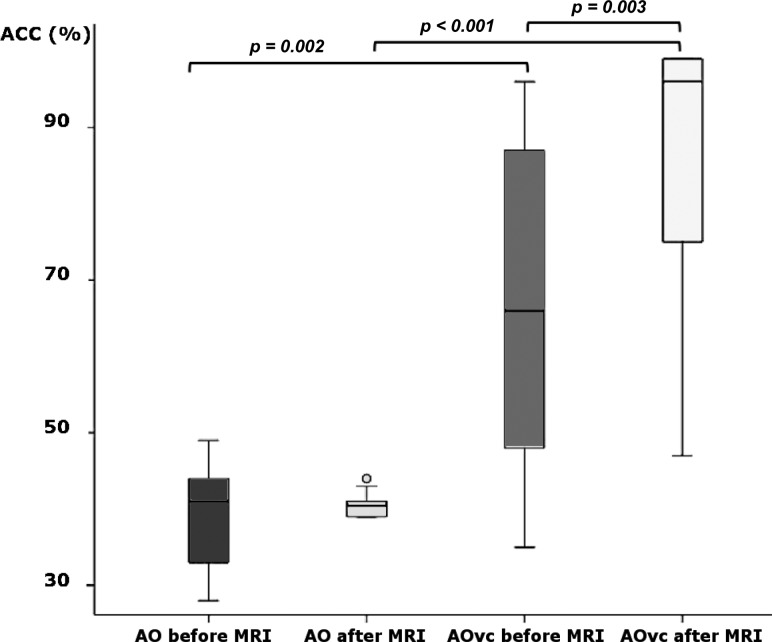
Group performance during action observation behavioral tasks. The box plot shows the relative accuracy (ACC) of force estimation in the different action observation (AO) and action observation with visual cue (AOvc) behavioral sessions (before and after the fMRI sessions). Significant differences between conditions are indicated (paired *t*-test).

**Figure 3.**
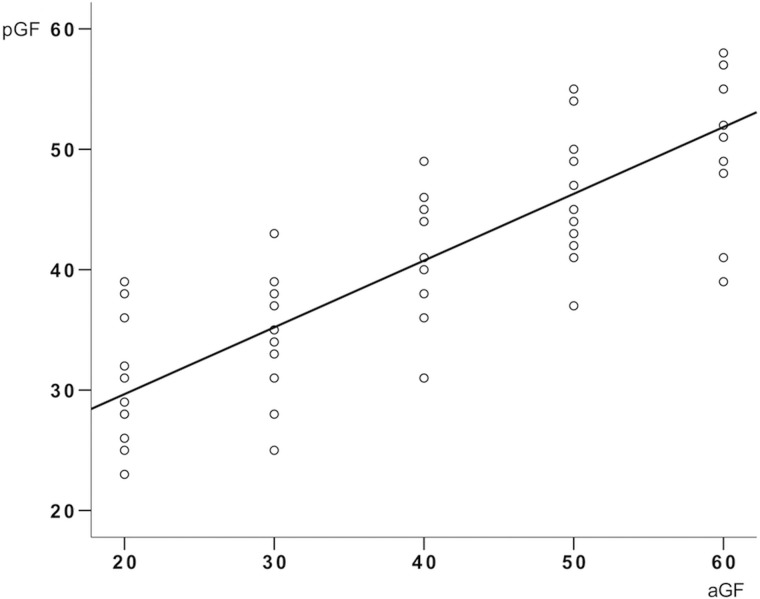
Relationship between applied and perceived grip force during the action observation behavioral task. The plot shows the relationship between grip force (GF) actually applied (aGF, i.e., GF presented on the video) and GF perceived (pGF, i.e., by the subjects watching the video) during the behavioral action observation (AO) task performed before and after fMRI recordings. The circles are individual subject responses and the line represents the group mean performance. A significant positive correlation was found between aGF and pGF (*R*^2^ = 0.98, *P* = 0.005).

The behavioral performance, at group level, when watching the AO and AOvc videos, is shown in Fig. 2. The accuracy of the perceived grip force (pGF) significantly differed between AO and AOvc, both before (*P* = 0.002) and after (*P* = 0.00008) the MRI session. Within the AOvc condition, pGF accuracy was higher after MRI (*P* = 0.003), while no significant differences were found in the AO condition (*P* = 0.955). As expected, GF recognition was higher in AOvc than AO (mean accuracy ± SD, 76 ± 22 and 39 ± 7, respectively), although at the end of the experiment, some subjects reported that the bar indicating levels of force (the visual cue) had not influenced their behavioral responses during AOvc. AO data showed a positive correlation between aGF and pGF (*r*^2^ = 0.98, *P* = 0.005) (Fig. 3), thus, confirming that the subjects were able to infer the actor’s movement in quantitative terms. Given that subjects correctly perceived the strength of the applied GF also when watching the AO video without the visual cue, subsequent analysis of fMRI data concerned only the AE and AO conditions.

### Whole Brain—BOLD Effects

**Figure 4.**
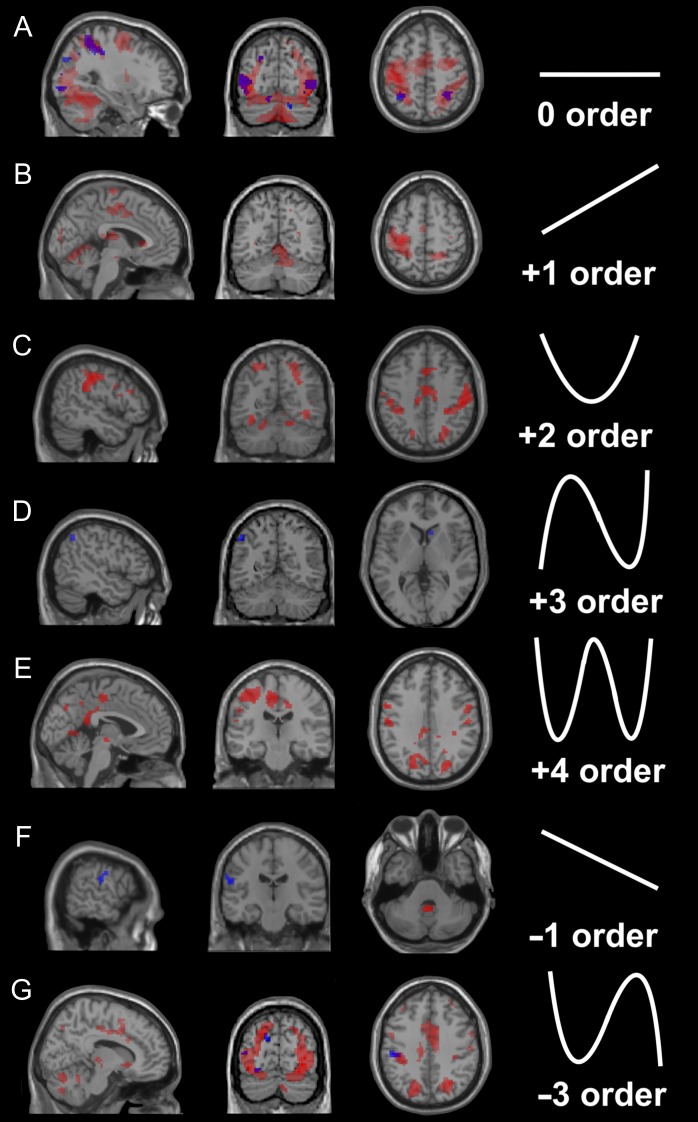
Whole-brain BOLD effects in action execution and observation. Brain maps at the group level corresponding to different orders of the BOLD-GF association in the action execution (AE, in red) and action observation (AO, in blue) conditions. The images show areas of activation at different orders. Note that both force-related and unrelated BOLD effects are found in the cerebral cortex and cerebellum. A threshold *P_u_*< 0.001 (*k* ≥ 10; *P_u_* = *P* uncorrected for multiple comparisons) was used for display purposes. The shape of the orthogonalised polynomial function that was fitted to the signals is shown for the (A) 0, (*B*) +1st, (*C*) +2nd, (*D*) +3rd, (*E*) +4th, (*F*) −1st, and (*G*) -3rd orders, to the right of the corresponding image showing significant clusters. In this and all the following figures, right is right and left is left.

Regionally specific effects for 0th (main effect), linear (+1st, −1st) and nonlinear (+2nd, +3rd, +4th, -3rd) order responses were detected, using whole-brain analysis, on AE and AO. Full data with figures and tables detailing significant effects (including coordinates, *T* values and cluster extents) are provided as supplementary material. AE activated many more regions than AO (Fig. 4), while both experimental conditions elicited regionally specific effects at 0th, −1st, and −3rd orders. Specifically, AO induced effects not only at the 0th order, but also at +3rd, 1st, and −3rd orders. These results reflect the presence of FRE during action observation.

### Whole Brain Conjunction

#### AE and AO Main Effect (0th Order)

**Figure 5.**
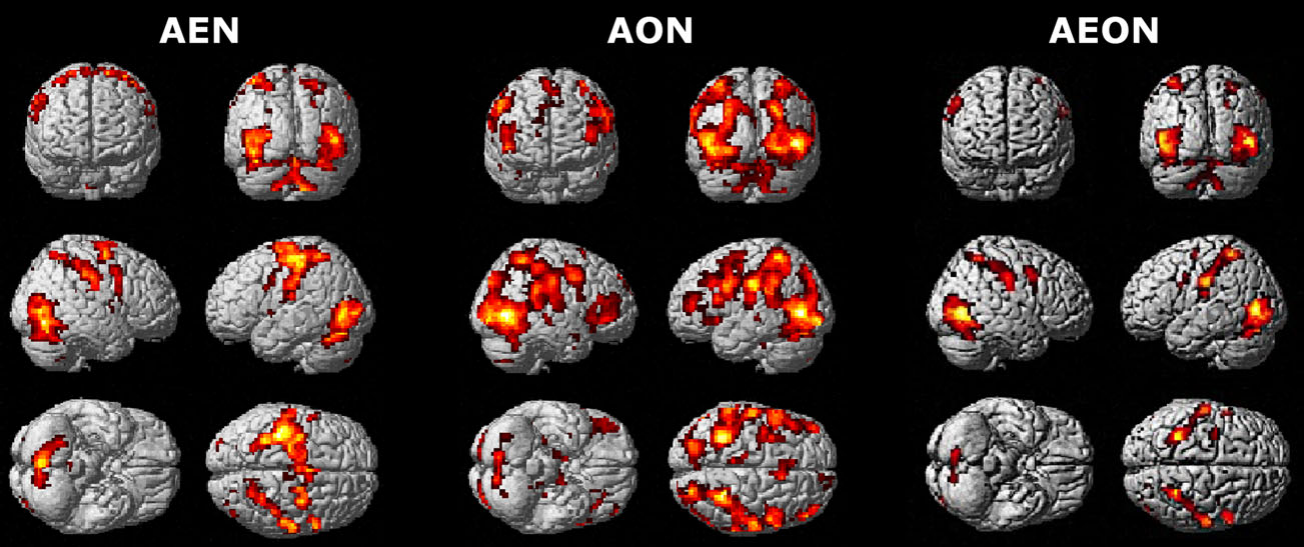
AEON: BOLD main effect. 3D whole-brain renderings of the main effects (0th order) in action execution (AEN), action observation (AON) and action execution–observation (AEON) networks. Note, in the AEON, the considerable overlap of effects in both the cerebral cortex and the cerebellum. Different thresholds were used for the 3 maps: *P_u_* < 0.0001 (*k* ≥ 10; *P_u_* = *P* uncorrected for multiple comparisons) was used for the AE condition; *P_u_* < 0.05 (*k* ≥ 10; *P_u_* = *P* uncorrected for multiple comparisons) was used for the AO condition; and *P* < 0.05 (*k* ≥ 10), with a small volume correction applied to the AO map, was used to obtain the AEON.

Several areas belonged to the extended AEON (in terms of a conjunction of zero order effects), and they included the occipital and temporal lobes, inferior and superior parietal cortices, precentral and postcentral gyri, inferior frontal gyrus, insula, thalamus and cerebellum. The occipitotemporal cluster extended into the cerebellar lobules VI and VII and cerebellar Crus I (Fig. 5 and Table 1).

**Table 1:** AEON: main effect (0th order)

Cluster	Peak					
Ext	*T*	*x*	*y*	*z*	Anatomical region	BA/Loc (%)
1986	8.64	51	−67	−2	R Middle Temporal Gyrus*	hOc4la (51)
	8.23	−45	−73	2	L Middle Occipital Gyrus	hOc5 [V5/MT] (53)
	6.83	−42	−79	−5	L Inferior Occipital Gyrus	hOc4la (69)
534	6.6	−30	−49	55	L Inferior Parietal Lobule	7 PC (SPL) (31)
	6.12	−54	−22	28	L Postcentral Gyrus	PFt (IPL) (46)
439	6.3	30	−49	58	R Superior Parietal Lobule	7PC (SPL) (40)
	5.59	−34	−34	43	R Postcentral Gyrus	3a (25)/1 (35)
	4.7	57	−16	37	R Postcentral Gyrus	
76	5.32	60	8	19	R Precentral Gyrus	44 (50)
33	4.61	−57	5	31	L Precentral Gyrus	44 (10)
30	4.46	−36	−4	16	L Insular Lobe	
13	4.45	24	−70	37	R Superior Occipital Gyrus	
	3.26	21	−79	43	R Cuneus	7P (SPL) (9)
46	3.41	−33	−7	64	L Precentral Gyrus	
35	3.11	−9	−10	7	L Thalamus	Thal: Prefrontal (86)

The table reports all the action execution–observation network (AEON) regions that presented a main effect (0th order). The statistical threshold is set using *P* < 0.05 (*k* ≥ 10) at the cluster level. Regions (*) survived a *P* < 0.05 correction for multiple comparisons at the peak level. The first cluster (ext = 1986) contains cerebellar activations (Crus 1 and lobules VI and VII). The last column reports the probability (expressed as a percentage) of these voxels being located in the respective Brodmann area (BA) or specific location (Loc) according to the cytoarchitectonic maps. ext = extension (number of voxels in a cluster); *T* = *T*-value at the voxel level. *x*, *y*, *z* are peak coordinates in MNI space (mm).

#### Force-Related Effects (Linear and Nonlinear Orders)

**Figure 6.**
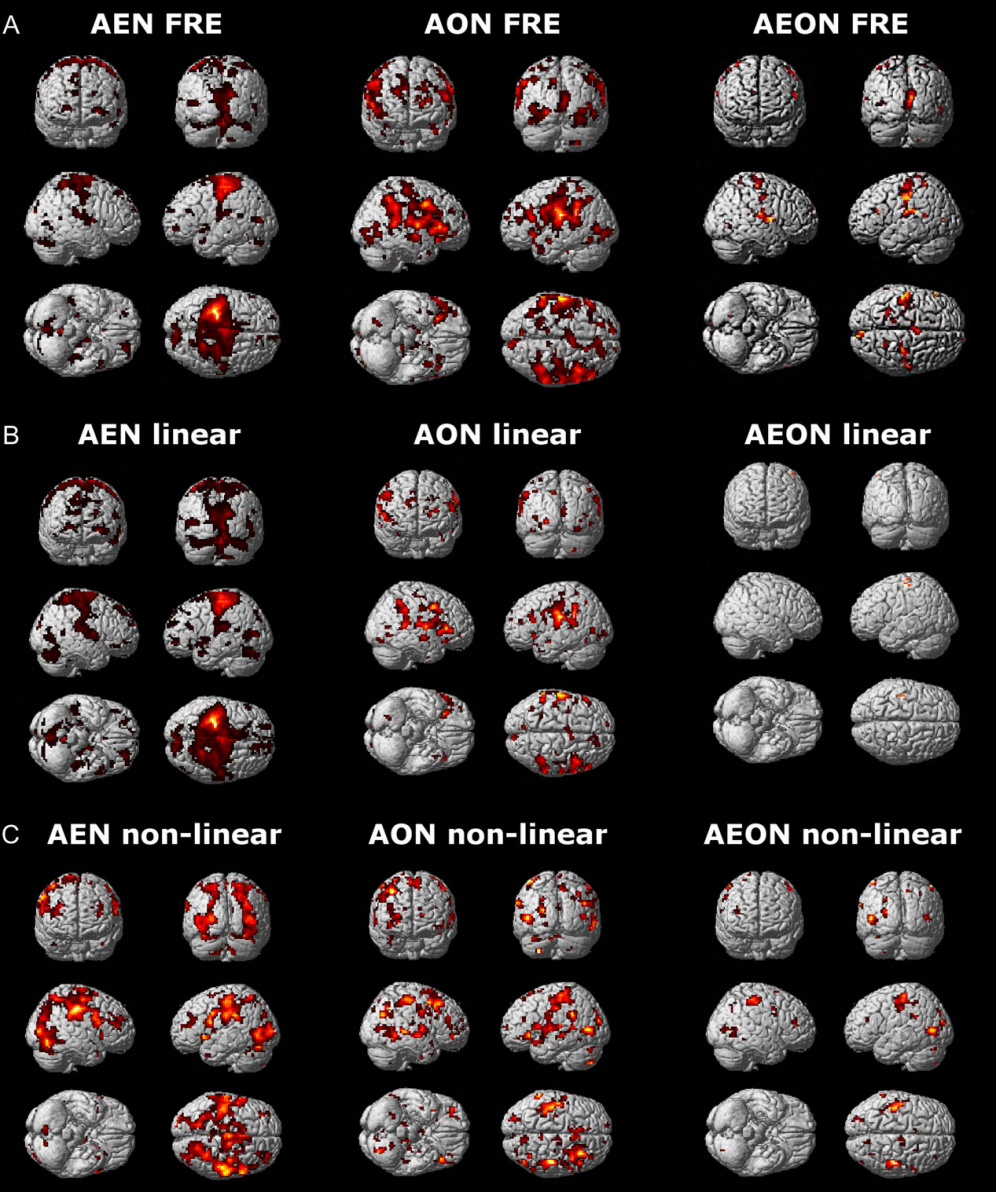
AEON: force-related BOLD effects. 3D whole-brain renderings of the force-related effects: (*A*) force-related effects (FRE), (*B*) linear force-related effects (linear FRE), and (*C*) nonlinear force-related effects (nonlinear FRE) in action execution (AEN), action observation (AON) and action execution–observation (AEON) networks. Note, in AEON, the prevalence of nonlinear associations in both the cerebral cortex and the cerebellum. Different thresholds were used for the 3 maps: *P_u_* < 0.0001 (*k* ≥ 10; *P_u_* = *P* uncorrected for multiple comparisons) was used for the action execution condition; *P_u_* < 0.05 (*k *≥ 10; *P_u_* = *P* uncorrected for multiple comparisons) was used for the action observation condition; and *P* < 0.05 (*k *≥ 10), with a small volume correction applied to the AO map, was used to obtain the AEON.

FREs (Fig. 6*A* and Table 2) were jointly expressed in several brain regions: supramarginal gyrus, calcarine gyrus, temporal gyrus, parietal lobe, insula, postcentral and precentral gyri, inferior frontal gyrus, middle cingulate cortex, posterior–medial and superior frontal cortices, rolandic operculum, lingual gyrus, basal ganglia, and cerebellum. A limited number of brain regions, that is, the precentral and postcentral gyri (Fig. 6*B*), exhibited a linear FRE (+1st and −1st orders) in both AE and AO. The most prevalent joint FREs were nonlinear (+2nd, +3rd, +4th, −2nd, −3rd, −4th orders) and identified in the: supramarginal gyrus, angular gyrus, precentral and postcentral gyri, occipital lobe, inferior and middle temporal gyri, rolandic operculum, inferior and superior parietal cortices, inferior and middle frontal gyri, middle cingulate cortex, insula, thalamus and cerebellum (Fig. 6*C*).

**Table 2:** AEON: force-related BOLD effects

Cluster	Peak					
ext (*k *≥ 10)	*T*	*x*	*y*	*z*	Anatomical region	BA/Loc (%)
62	6.79	−3	20	−2	L Caudate Nucleus	33 (36)
340	23.39	−54	−22	22	L Supramarginal Gyrus*	OP1 [SII] (53)
	12.92	−45	−34	19	L Superior Temporal Gyrus*	PFcm (IPL) (56)
	11.22	−48	−28	49	L Inferior Parietal Lobule*	2 (54)
	10.11	−36	−16	10	L Insular Lobe*	Ig2 (56)
	9.58	−57	−16	43	L Postcentral Gyrus*	1 (57)
	8.5	−51	−22	40	L Inferior Parietal Lobule*	2 (46)
	6.97	−51	−16	31	L Postcentral Gyrus*	3b (59)
	5.2	−27	−22	70	L Precentral Gyrus	
15	15.61	−57	26	22	L Inferior Frontal Gyrus (p. Triangularis)*	45 (64)
	9.16	−54	29	25	L Inferior Frontal Gyrus (p. Triangularis)*	45 (38)
226	15.41	9	2	52	R Posterior–Medial Frontal*	
	12.75	9	8	40	R Middle Cingulate Cortex*	
	8.38	0	−1	58	L Posterior–Medial Frontal*	
	7.62	−6	−10	55	L Posterior–Medial Frontal*	
	6.56	3	−19	49	R Posterior–Medial Frontal	4a (17)
	3.97	0	−19	37	L Middle Cingulate Cortex	
	2.91	3	−10	34	R Middle Cingulate Cortex	
143	13.76	54	−7	10	R Rolandic Operculum*	OP4 [PV] (44)
	13.39	60	2	10	R Rolandic Operculum*	44 (11)
	11.65	66	−16	25	R SupraMarginal Gyrus*	PFop (IPL) (39)
	7.8	51	−19	16	R Rolandic Operculum*	OP1 [SII] (64)
	6.56	66	−19	13	R Superior Temporal Gyrus	TE 3 (31)
	5.93	45	−28	16	R Superior Temporal Gyrus	OP1 [SII] (41)
	3.57	57	−31	22	R Superior Temporal Gyrus	PFcm (IPL) (40)
31	11.65	−3	−64	4	L Lingual Gyrus*	hOc1 [V1] (51)
185	10.22	6	−91	16	R Cuneus*	hOc2 [V2] (74)
	9.27	6	−94	10	R Cuneus*	hOc2 [V2] (73)
	6.8	18	−73	4	R Calcarine Gyrus	hOc1 [V1] (75)
	5.58	15	−79	1	R Lingual Gyrus	hOc1 [V1] (65)
10	7.85	39	−19	43	R Precentral Gyrus	4p (54)
24	7.14	15	−64	−26	R Cerebellum*	Lobule VI (Hem) (77)
25	6.84	−12	−61	−17	L Cerebellum	Lobule VI (Hem) (94)
13	6.65	21	−49	−23	R Cerebellum	Lobule VI (Hem) (77)
38	6.42	−33	−46	67	L Superior Parietal Lobule	1 (7)
	5.34	−24	−49	61	L Superior Parietal Lobule	5 L (SPL) (51)
33	6.41	60	−13	43	R Postcentral Gyrus	1 (58)
	3.01	51	−10	52	R Precentral Gyrus	4a (8)
16	6.38	54	−67	−5	R Inferior Temporal Gyrus	hOc4la (68)
	2.97	48	−73	4	R Middle Occipital Gyrus	hOc4la (72)
97	6.25	30	−25	61	R Precentral Gyrus	4p (7)
	5.84	48	−22	61	R Postcentral Gyrus	1 (22)
	5.42	51	−25	58	R Postcentral Gyrus	1 (82)
	4.56	21	−25	73	R Precentral Gyrus	4a (20)
12	6.21	−33	−13	22	L Insular Lobe	
15	6.16	−39	−76	7	L Middle Occipital Gyrus	hOc4la (26)
10	5.82	15	41	1	R Anterior Cingulate Cortex	
23	4.8	21	−49	61	R Superior Parietal Lobule	5 L (SPL) (50)
	4.66	27	−52	67	R Superior Parietal Lobule	7PC (SPL) (81)
25	4.04	−15	−37	70	L Postcentral Gyrus	4a (26)
	3.57	−18	−40	76	L Postcentral Gyrus	1 (16)
13	3.66	15	−46	43	R Precuneus	5 Ci (SPL) (7)
	3.19	12	−46	49	R Precuneus	5 Ci (SPL) (37)

The table reports all the action execution–observation network (AEON) regions that presented a force-related effect (FRE). The statistical threshold is set using *P* < 0.05 (*k *≥ 10) at the cluster level. Regions (*) survived a *P* < 0.05 correction for multiple comparisons at the peak level. The last column reports the probability (expressed as a percentage) of these voxels being located in the respective Brodmann area (BA) or specific location (Loc) according to the cytoarchitectonic maps. ext=number of voxels in a cluster; *T* = *T*-value at the voxel level. *x*, *y*, *z* are the peak coordinates in MNI space (mm).

### SUIT—BOLD Effects

**Figure 7.**
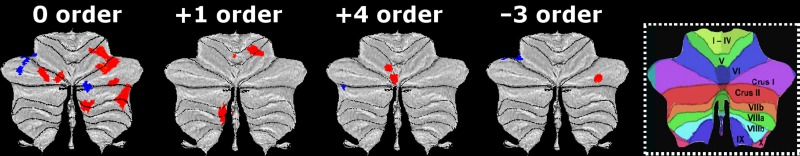
Cerebellar BOLD effects in action execution and observation. SUIT flat maps at the group level corresponding to different orders of the BOLD–GF association in action execution (AE) and action observation (AO) conditions. Note the force-related and main (0th order) BOLD effects in different cerebellar areas. In the images, areas of activation at different orders of effect are shown for both AE (in red) and AO (in blue). A SUIT flat map with labels of cerebellar lobules is shown in the bottom right corner. A threshold *P_u_* < 0.001 (*k *≥ 10; *P_u_*= *P* uncorrected for multiple comparisons) was used for display purposes.

The AE condition detected more activated cerebellar regions than the AO one did (Fig. 7). In AE cerebellar specific effects were detected in lobule V and VIII (+1st order), lobule VI (0th and +4th orders), lobule VII (0th order), and Crus I (0th, +4th and −3rd orders). AO effects were observed in lobule VI (0th and −3rd orders), Crus I (0th order), and Crus II (+4th order).

### SUIT—Conjunction

#### AE and AO Effect (0th Order)

**Figure 8.**
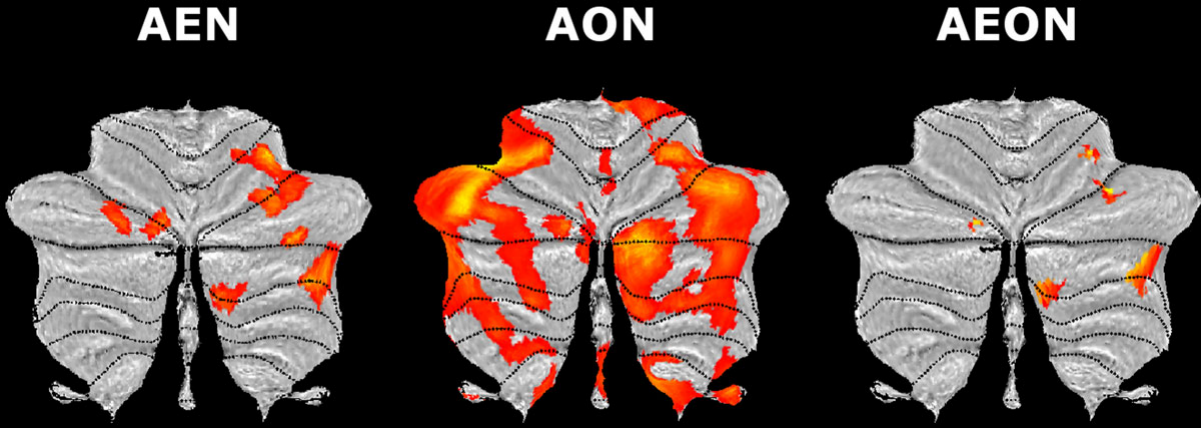
Cerebellar component of AEON: main BOLD effects. SUIT flat maps of the main effects (0th order) in the cerebellar component of the action execution (AEN), action observation (AON) and action execution–observation (AEON) networks. Note, in AEON, the extended involvement of posterior and lateral areas of the cerebellum. Different thresholds were used for the 3 maps: *P_u_*< 0.001 (*k* ≥ 10; *P_u_* = *P* uncorrected for multiple comparisons) was used for the action execution condition; *P_u_* < 0.05 (*k* ≥ 10; *P_u_* = *P* uncorrected for multiple comparisons) was used for the action observation condition; and *P* < 0.05 (*k* ≥ 10), with a small volume correction applied to the AON map, was used to obtain the AEON.

Lobules VI and VII and Crus I and II were jointly involved in AE and AO (Fig. 8).

#### Force-Related Effects (Linear and Nonlinear Orders)

**Figure 9.**
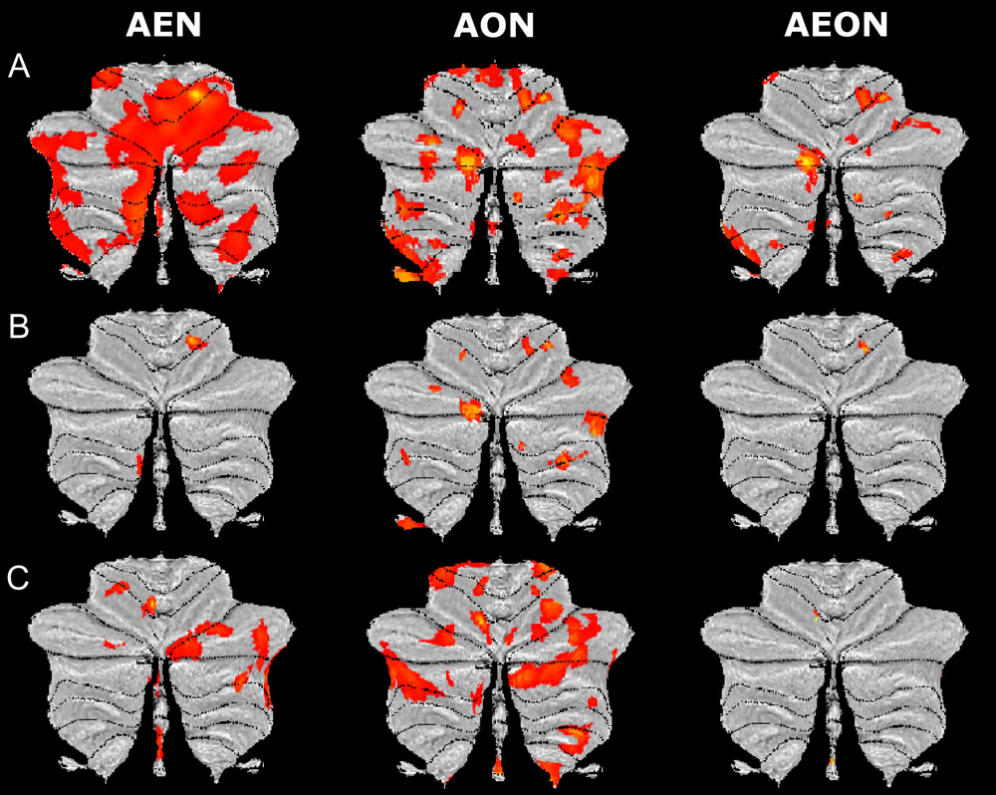
Cerebellar component of AEON: force-related BOLD effects. SUIT flat maps of the (*A*) force-related effects (FRE), (*B*) linear force-related effects (linear FRE), and (*C*) nonlinear force-related effects (nonlinear FRE) in the cerebellar component of the action execution (AEN), action observation (AON) and action execution–observation (AEON) networks. Note, in AEON, the distribution of force-related BOLD effects over several cerebellar areas in the anterior and posterior cerebellum. Different thresholds were used for the 3 maps: *P_u_* < 0.001 (*k* ≥ 10; *P_u_*= *P* uncorrected for multiple comparisons) was used for the action execution condition; *P_u_* < 0.05 (*k *≥ 10; *P_u_*= *P* uncorrected for multiple comparisons) was used for the action observation condition; and *P* < 0.05 (*k* ≥ 10), with a small volume correction applied to the AON map, was used to obtain the AEON.

FREs were jointly identified in: Crus I and lobules V and VI (Fig. 9*A*). A cluster in lobule V presented a linear FRE (+1st and −1st orders) while a nonlinear FRE (+2nd, +3rd +4th, −2nd, −3rd, −4th orders) was found in 2 clusters in lobule VI and IX (Fig. 9*B* and *C*, respectively).

## Discussion

In this study, we report for the first time the existence of force-related BOLD effects in an extended AEON involving cerebral and cerebellar regions that are both motor and associative in nature. These regions not only respond to observed and executed actions, but also share patterns of linear and nonlinear BOLD responses to parametric variations in GF. Linear BOLD–GF associations occurred in motor regions, while nonlinear BOLD–GF associations were found in regions specific to somatosensory state estimation, motor simulation and cognitive control. The cerebellum was found to be a key structure within the AEON, showing regional-specific correlations with force. These effects support the concept that the AEON extends to the cerebellum and to a set of cortical regions that are critical for imitation learning. The results are discussed and integrated with our current understanding of brain function in terms of the intrinsic functional connectivity of 7 fundamental networks (visual, somatomotor, ventral and dorsal attention, frontoparietal, limbic and default networks) (Yeo et al. 2011).

### Behavioral Performance and Learning Effects

The subjects were found to be able to evaluate visually the efforts of others. There are 3 considerations indicating that this ability was independent of learning during the test (Weigelt et al. 2008). First, in order to saturate learning, all the subjects underwent AO training before the fMRI experimental sessions; this training is known to facilitate motor learning (Stefan et al. 2005; Salama et al. 2011) and increase force production by optimizing motor neuron recruitment (Porro et al. 2007). Second, the order of presentation of the AE, AO, AOvc sessions was randomized, thus limiting a potential variability in attentional load (e.g., induced by fatigue). Third, the accuracy in force detection was higher in AOvc than in the AO sessions. Therefore, independently of subject performance, the visual cue has a facilitator effect with respect to naturalistic stimulation (i.e., actual movement and changes in body parts during action), but the latter is nonetheless sufficient to perceive the intensity of another’s movements.

It should be noted that in the AOvc condition we detected a learning effect, implying a facilitation of force recognition along trials. Moreover, we detected a higher variability of performance between participants in the AOvc compared with the AO condition; this might be due to attention being focused either on the visual cue or on the hand itself. However, the debriefing at the end of the experiment indicated that several participants had ignored the bar. For these reasons, we did not further consider the AOvc condition in our analysis.

### BOLD Effects Elicited by AE and AO

The BOLD effects elicited by AE occurred mostly in areas directly involved in motor planning, execution, and control (Alahmadi, Samson et al. 2015) (for a detailed description see Table 3). The main effect of AE was observed in premotor and sensorimotor cortices as well as in parietal, occipital and cerebellar cortices devoted to global sensorimotor processing (Neely et al. 2013) and included in visual, dorsal attention and somatomotor networks, while a linear activation with force was found in primary motor cortex and anterior cerebellum, that actually do encode force (Keisker et al. 2009) and belong to the somatomotor network. Nonlinear relationships with GF were found in parietal, frontal, cingulate and insular cortices, and in thalamus, basal ganglia and cerebellum, which form large-scale loops involved in the control of fine precision grip forces and motor learning (Graybiel 1998; Ehrsson et al. 2001; Kuhtz-Buschbeck et al. 2001). These loops could be linked with visual, ventral and dorsal attention, frontoparietal, somatomotor and default networks. Minor differences with previous squeeze ball studies (Alahmadi, Samson et al. 2015) could be related to the higher complexity of the present paradigm (which included behavioral training sessions and 3 different tasks).

**Table 3:** Summary of BOLD effects for AEN, AON, and AEON

Areas	AE			AO			AEON			Functions	References
	0^th^	L	N	0^th^	L	N	0^th^	L	N		
**Occipital**											
**Cuneus**		#				§	°			Visual analysis of action/detection of biological motion/visual mental imagery of hand gesture/object recognition	(Dupont et al. 1994; Hermsdörfer et al. 2001; Knauff et al. 2002; Rizzolatti and Craighero 2004; Wiggett and Downing 2011; Molenberghs et al. 2012; Romaiguère et al. 2014; Ishibashi et al. 2016)
**Fusiform OG**			§	°		§			§		
Lingual OG			§								
**Inferior OG**	°			°			°				
**Middle OG**	°		§	°		§	°		§		
**Superior OG**			§	°		§	°		§		
**Temporal**											
**Inferior TG**			§						§	Visual motion processing/experience and observation of touch	(Beer et al. 2002; Blakemore et al. 2005)
**Middle TG**				°	#	§			§		
Superior TG		#	§								
Hippocampus		#									
**Parietal**											
**Postcentral PG**	°	#	§	°	#		°	#	§	AE, AO and imitation/movement plan perception/proprioceptive representations/tactile simulation/spatial attention	(Buccino et al. 2001; Grèzes and Decety 2001; Jovicich et al. 2001; Tanaka et al. 2001; Knauff et al. 2002; Gazzola and Keysers 2009; Shomstein 2012)
Precuneus			§								
**Inferior PG**				°		§	°		§		
**Superior PG**	°	#	§	°			°		§		
**Supramarginal G**	°		§	°	#				§		
Angular G									§		
**Frontal**											
**Precentral G**	°	#	§	°		§	°	#	§	Mirror neurons/response inhibition/attention/inhibition of motor execution/motor goal/hand-object integration/motor planning/working memory	(Cheng et al. 1995; Fagg and Arbib 1998; Shima and Tanji 1998; Brass et al. 2001; Grèzes and Decety 2001; Menon et al. 2001; Leung et al. 2002; Kasess et al. 2008; Morin and Grèzes 2008; Nakata et al. 2008; Caligiore et al. 2013)
Inferior FG			§		#				§		
**Middle FG**			§			§			§		
**Superior FG**		#	§						§		
Orbital FG		#									
**Deep GM**											
**Thalamus**	°	#	§				°		§	Modulation of movement, motivation and reward/modulation of motor preparation/force amplitude generation and prediction	(Spraker et al. 2007; Wasson et al. 2010; Braadbaart et al. 2013; Caligiore et al. 2013)
Putamen			§								
Pallidum			§								
Caudate			§			§					
Amygdala		#				§					
**Others**											
Basal Forebrain		#	§							Maintenance of spatial attention during goal-directed actions/action style processing/sensorimotor integration/preparatory suppression of imitative responses	(Luks and Simpson 2004; Cauda et al. 2012; Leech et al. 2012; Di Cesare et al. 2016; Campbell et al. 2018)
Brainstem						§					
ACC			§								
**MCC**	°	#	§						§		
PCC			§								
**Insula**			§	°	#		°		§		
**Rolandic op**			§						§		
**Cerebellum**											
**Lobule V**		#						#		Motor learning/observation of movements/control for movement execution/force amplitude generation and prediction/somatosensory state estimation/cognitive control	(Decety et al. 1997; Grèzes and Decety 2001; Spraker et al. 2007; Gazzola and Keysers 2009; Molenberghs et al. 2012; Stoodley et al. 2012; Buckner 2013; E KH et al. 2014; Weaver 2015)
**Lobule VI**			§	°		§	°		§		
**Lobule VII**	°		§				°				
Lobule VIII		#									
**Crus 1**	°		§	°			°				
**Crus 2**						§	°				
**Lobule IX**									§		

The table reports all the action execution (AEN), action observation (AON), and action execution–observation (AEON) networks areas that showed main (0= 0th order) or force-related (L=linear, N = nonlinear) BOLD–GF relationships. The brain areas are listed and grouped by region: cerebral lobes (occipital, temporal, parietal, and frontal), deep GM (gray matter), others and cerebellum. Three different symbols (°, #, §) are used to indicate, respectively, the presence of main, linear or nonlinear effects in the corresponding area. Functions and references on functions are reported for each group of regions. It should be noted that the thresholds used in the fMRI analysis differ depending on the condition (AE or AO) (see Materials and Methods). It has to be highlighted that the threshold problem is irrelevant for the conjunction anaylsis (AEON) columns because it refers to an independent analysis on overlapping. Bold type in the “areas” columns has been used to facilitate the location in the table of regions resulted from the conjunction analysis. G=Gyrus; OG=Occipital Gyrus; TG=Temporal Gyrus; PG=Parietal Gyrus; FG=Frontal Gyrus; op = operculum. ACC = Anterior Cingulated Cortex, MCC = Middle Cingulated Cortex, PCC = Posterior Cingulate Cortex.

The BOLD effects elicited by AO were recorded in an extended and distinctive set of occipital, parietal and frontal regions involved in motor but also sensory and cognitive processing (for a detailed description see Table 3). The main effect of AO was observed in the occipital and parietal regions related to motion perception and attention (Becker-Bense et al. 2012; Thompson and Parasuraman 2012) that are considered to be part of the visual network and the ventral and dorsal attention networks. Linear responses with respect to GF occurred mainly in the postcentral gyrus, involved in processing proprioceptive and tactile representations of the manipulated object (Ebisch et al. 2008) and included in the somatomotor network. Nonlinear responses with respect to GF occurred in occipital and temporal cortices and inferior parietal lobule, involved in object recognition (Reed et al. 2004), inhibition of movement (Menon et al. 2001), spatial focusing of attention (Ptak 2012), and intention understanding (Koul et al. 2018). These functions are supported by the inclusion of activated regions in the visual, dorsal attention and default networks. These components, either main or FREs, probably provide the substrate for the interpretation and simulation of the actions of others (McGregor and Gribble 2015), while actual movement is inhibited.

The detection of specific nonlinear force-related BOLD effects in areas involved in AE or AO could imply complex task-related interplay of different neuronal populations (e.g., inhibitory and excitatory) within local networks. Understanding the biophysical basis of such nonlinearities will need realistic models and further investigation of neurovascular coupling under different conditions.

### The Common Neural Substrate of AE and AO

The AEON, identified as the voxels shared by AEN and AON, was observed in cerebral cortex, thalamus, basal ganglia, and cerebellum (Christensen et al. 2014) (for a detailed description see Table 3). The main effect of AEON was observed in occipital cortex, middle temporal gyrus, precentral and postcentral gyri, inferior and superior parietal lobules, insula, thalamus, and posterior cerebellum. These areas are involved in visual imagery of hand gestures, including visuospatial and motion processing (Hermsdörfer et al. 2001; Knauff et al. 2002), forward/inverse control for movement planning and execution, action style processing (Cauda et al. 2012), inhibition of motor execution to prevent imitative responses (Kilner 2011). These are, indeed, the fundamental ingredients of motor planning based on observation of actions (Caligiore et al. 2013). The activation of inferior and superior parietal lobules (which are part of the core mirror network) (Molenberghs et al. 2012) with occipital cortex, precentral and postcentral gyri and insula, suggest that AEON includes components from the visual, mirroring/somatomotor, ventral and dorsal attention networks.

The AEON areas showing FREs were also extensively distributed in occipital cortex, superior temporal gyri, inferior and superior parietal lobules, precentral and postcentral gyri, inferior frontal gyrus, medial frontal gyrus, precuneus, cingulate gyrus, caudate, thalamus, and cerebellum. These areas are involved in the experience and observation of touch (Blakemore et al. 2005), maintenance of spatial attention during goal-directed actions (Wasson et al. 2010; Cauda et al. 2012; Leech et al. 2012), sensorimotor integration, and force amplitude generation and prediction (Grèzes and Decety 2001; Blakemore et al. 2005). Moreover, the medial frontal gyrus is considered, with the temporoparietal junction, the “core network” of attribution of mental states (Schurz et al. 2014; Molenberghs et al. 2016) while the precuneus has been proposed to have a role in mental imagery to represent others’ perspective (Cavanna and Trimble 2006). Therefore, FREs are particularly important in conferring the ability to detect the effort of others not just requiring the intervention of the mirroring/somatomotor, ventral and dorsal attention and frontoparietal networks but also of the mentalizing/default network.

Interestingly, activation in the AEON areas was mostly nonlinear, while linear relationships with GF were found only in a restricted part of the precentral and postcentral cortices (Gazzola and Keysers 2009) that are included in the mirroring/somatomotor network. Therefore, the AEON is engaged mainly in a nonlinear fashion during force processing.

### Cerebellar Involvement in AEON

The cerebellum is known to operate as a generalized forward controller (D’Angelo and Casali 2012) that aids motor planning by predicting the sensory consequences of a motor act, such that a motor plan is coded in terms of an anticipatory sensory state (Blakemore, Goodbody et al. 1998; Blakemore, Wolpert et al. 1998; Callan et al. 2013). In the present context, the sensory state would be provided by AO, and motor predictions would be based on internal cerebellar representations of the system (body and muscle) state (Diedrichsen et al. 2010; Shadmehr and Mussa-Ivaldi 2012). Thus, the cerebellum is well geared for simulating movements after receiving information about the movement of others, in terms of the appropriate sequence (timing) and force (gain) (Yamazaki and Nagao 2012; Callan et al. 2013).

The cerebellum is strongly interconnected with the cerebral cortex through 7 fundamental resting-state networks (Yeo et al. 2011). In particular, Buckner et al. (2011) clearly identified in the cerebellum distinct mirroring and mentalizing networks (as part of the larger somatomotor and default networks respectively) that were directly connected to homolog networks in the cerebral cortex. Confirming this network structure, Van Overwalle and colleagues reinterpreted their initial meta-analysis (Van Overwalle et al. 2014) in terms of this network structure and found strong evidence for it (Van Overwalle, Baetens et al. 2015). In addition, a meta-analytical connectivity analysis revealed a strong distinction between anterior mirroring and posterior mentalizing areas in the cerebellum linked to classic mirror and mentalizing areas in the cerebral cortex (Van Overwalle, D’aes et al. 2015). This was confirmed by functional connectivity studies relating mentalizing and executive control functioning to the cerebellum (Van Overwalle and Mariën 2016). Therefore, the cerebellum could also be involved in predicting the consequences and scope of other’s actions by reconstructing hypothetical events (Buckner et al. 2011; Van Overwalle and Mariën 2016).

It should be noted that, in our study, cerebellar responses were embedded in a larger cluster that included occipitotemporal areas. The combined activation in the AEON in lobules VI and VII and Crus I and II could be considered part of the ventral and dorsal attention, frontoparietal, and mentalizing/default networks compounded by the mirroring/somatomotor network from specific FREs (Buckner et al. 2011). Indeed, the linear FRE in lobules V, part of the mirroring/somatomotor network, reveals the involvement of cerebellum in motor functions (Glickstein et al. 1994; Schmahmann 1996; Stoodley et al. 2012). The nonlinear effect in lobules VI and IX, part of both mirroring/somatomotor and mentalizing/default networks, would suggest the cerebellar involvement in the integration of motor processing and cognitive/emotional control (Stoodley and Schmahmann 2010).

Altogether, these effects confirm the cerebellar involvement in both mirroring and mentalizing networks. Moreover, these patterns of linear and nonlinear responses in the cerebellar components of the AEON are consistent with results showing that motor-generating areas respond linearly with GF, while associative and cognitive areas have a more complex relationship with GF. These response profiles may reflect distributed responses, mediated by connections that have been characterized structurally and physiologically in rodents, primates, and humans (Schmahmann and Pandya 1995; Kelly and Strick 2003; Ramnani 2006; Strick et al. 2009; Watson et al. 2014; Palesi et al. 2015, 2017).

### High-Order Force–BOLD Relationships in the Cerebellum and Cerebral Cortex

While a monotonic relationship between BOLD response and GF levels was found in primary motor areas (M1 and anterior cerebellum)—and could be related to the increased neuronal recruitment with increasing GF (Cramer et al. 2002; Keisker et al. 2009)—nonlinearities were typically detected in areas implicated in multimodal integration and higher aspects of motor control (premotor, associative and sensory areas both in the cerebral cortex and the cerebellum), where a complex blend of signals converges to regulate motor output. It has been previously argued (Alahmadi, Samson et al. 2015) that second-order responses at intermediate force levels could be “metabolically optimal” and reflect more efficient processing in a motor regime requiring fewer corrective actions and less attention to sensory inputs. For example, nonlinearity may be due to fluctuation of attention levels modulating neural activity (Binkofski et al. 2002). However, it should be appreciated that it is difficult to make detailed neurophysiological inferences based exclusively on fMRI signals. For example, nonlinearities (including nonlinear neuronal responses, nonlinear engagement of local inhibitory circuits, nonlinear mapping from neuronal activity to haemodynamic responses and finally, nonlinearities associated with the haemodynamic response function generating T2* signals) could arise at a number of different levels (Friston et al. 2000; Mechelli et al. 2001). The engagement of the underlying neuronal circuits, both in the cerebral cortex and the cerebellum, may benefit from further investigation using repetition-suppression fMRI paradigms (Kilner 2011; Barron et al. 2016) or multivoxel pattern analysis (Turella et al. 2009) in conjunction with animal recordings and large-scale model simulations (D’Angelo and Gandini Wheeler-Kingshott 2017).

### Potential Limitations

Despite the coherent functional framework emerging from this investigation, the relatively small number of subjects may affect its statistical power in detecting active areas. Although previous studies used similar numbers of subjects, a larger sample may be beneficial to confirm our findings. However, it is important to note that significant results obtained using a small sample usually mean that the effect size is large (Flandin and Friston 2017). From a statistical point of view, the use of parametric models is an efficient way of accommodating nonlinear (neurometric) response functions within the established GLM framework (Friston 2012). However, given the concern that detection of active areas may have been reduced because of habituation due to multiple engagement in AO (Krekelberg et al. 2006), it would be useful to devise alternative paradigms in order to refine our AEON parametric characterization.

## Conclusions

The extended AEON identified in this fMRI study engages large-scale brain networks capable of remapping the visual detection of the actions, and also effort, of others onto the observer’s own motor system. These circuits, furnish not only understanding of other people’s goals, that is, the “mirror” effect (Rizzolatti and Craighero 2004), but also the building blocks of executive control, impacting on various aspects of motor planning and programming, working memory, selective attention, and behavioral inhibition (Rizzolatti and Craighero 2004; Calvo-Merino et al. 2006; Filimon et al. 2007; Thompson et al. 2007; Hamilton and Grafton 2008; Caspers et al. 2010; Cavallo et al. 2015). The cerebrocerebellar loops, using the motor system as a forward model (Miall 2003; Kilner et al. 2007; Ito 2008; D’Angelo and Casali 2012; Callan et al. 2013), could play a crucial role in sensorimotor prediction and internal simulation of movement. It has been suggested that the insular and cingulate cortices, activated in parallel to the sensorimotor loops, allow exteroception to be integrated with interoception (Bodegård et al. 2001; Craig 2003; Pineda et al. 2009) and external observational cues to be transformed into internal sensorimotor plans. The identification of this extended AEON as a plausible substrate for imitation learning could facilitate and improve the clinical application of action observation in neurorehabilitation (Porro et al. 2007; Garrison et al. 2013; Buccino 2014).

## Supplementary Material

Supplementary Data

## References

[bhy322C1] Alahmadi AA, Pardini M, Samson RS, D’Angelo E, Friston KJ, Toosy AT, Gandini Wheeler-Kingshott CA 2015 Differential involvement of cortical and cerebellar areas using dominant and nondominant hands: an FMRI study. Hum Brain Mapp. 36:5079–5100. 26415818 10.1002/hbm.22997 PMC4737094

[bhy322C2] Alahmadi AA, Pardini M, Samson RS, Friston KJ, Toosy AT, D’Angelo E, Gandini Wheeler-Kingshott CA 2017 Cerebellar lobules and dentate nuclei mirror cortical force-related-BOLD responses: beyond all (linear) expectations. Hum Brain Mapp. 38:2566–2579. 28240422 10.1002/hbm.23541 PMC5413835

[bhy322C3] Alahmadi AA, Samson RS, Gasston D, Pardini M, Friston KJ, D’Angelo E, Toosy AT, Wheeler-Kingshott CA 2015 Complex motor task associated with non-linear BOLD responses in cerebro-cortical areas and cerebellum. Brain Struct Funct. 221:2443–2458. 25921976 10.1007/s00429-015-1048-1 PMC4884204

[bhy322C4] Avanzino L, Bove M, Pelosin E, Ogliastro C, Lagravinese G, Martino D 2015 The cerebellum predicts the temporal consequences of observed motor acts. PLoS One. 10:e0116607. 25689858 10.1371/journal.pone.0116607 PMC4331528

[bhy322C5] Barron HC, Garvert MM, Behrens TE 2016 Repetition suppression: a means to index neural representations using BOLD? Philos Trans R Soc Lond B Biol Sci. 371:1–14. 10.1098/rstb.2015.0355 PMC5003856 27574308

[bhy322C6] Becker-Bense S, Buchholz HG, zu Eulenburg P, Best C, Bartenstein P, Schreckenberger M, Dieterich M 2012 Ventral and dorsal streams processing visual motion perception (FDG-PET study. BMC Neurosci. 13:81. 22800430 10.1186/1471-2202-13-81 PMC3467181

[bhy322C7] Beer J, Blakemore C, Previc FH, Liotti M 2002 Areas of the human brain activated by ambient visual motion, indicating three kinds of self-movement. Exp Brain Res. 143:78–88. 11907693 10.1007/s00221-001-0947-y

[bhy322C8] Binkofski F, Fink GR, Geyer S, Buccino G, Gruber O, Shah NJ, Taylor JG, Seitz RJ, Zilles K, Freund HJ 2002 Neural activity in human primary motor cortex areas 4a and 4p is modulated differentially by attention to action. J Neurophysiol. 88:514–519. 12091573 10.1152/jn.2002.88.1.514

[bhy322C9] Blakemore SJ, Bristow D, Bird G, Frith C, Ward J 2005 Somatosensory activations during the observation of touch and a case of vision-touch synaesthesia. Brain. 128:1571–1583. 15817510 10.1093/brain/awh500

[bhy322C10] Blakemore SJ, Goodbody SJ, Wolpert DM 1998 Predicting the consequences of our own actions: the role of sensorimotor context estimation. J Neurosci. 18:7511–7518. 9736669 10.1523/JNEUROSCI.18-18-07511.1998 PMC6793221

[bhy322C11] Blakemore SJ, Wolpert DM, Frith CD 1998 Central cancellation of self-produced tickle sensation. Nat Neurosci. 1:635–640. 10196573 10.1038/2870

[bhy322C12] Bodegård A, Geyer S, Grefkes C, Zilles K, Roland PE 2001 Hierarchical processing of tactile shape in the human brain. Neuron. 31:317–328. 11502261 10.1016/s0896-6273(01)00362-2

[bhy322C13] Braadbaart L, Williams JH, Waiter GD 2013 Do mirror neuron areas mediate mu rhythm suppression during imitation and action observation? Int J Psychophysiol. 89:99–105. 23756148 10.1016/j.ijpsycho.2013.05.019

[bhy322C14] Brass M, Zysset S, von Cramon DY 2001 The inhibition of imitative response tendencies. Neuroimage. 14:1416–1423. 11707097 10.1006/nimg.2001.0944

[bhy322C16] Buccino G 2014 Action observation treatment: a novel tool in neurorehabilitation. Philos Trans R Soc Lond B Biol Sci. 369:20130185. 24778380 10.1098/rstb.2013.0185 PMC4006186

[bhy322C17] Buccino G, Binkofski F, Fink GR, Fadiga L, Fogassi L, Gallese V, Seitz RJ, Zilles K, Rizzolatti G, Freund HJ 2001 Action observation activates premotor and parietal areas in a somatotopic manner: an fMRI study. Eur J Neurosci. 13:400–404. 11168545

[bhy322C18] Büchel C, Holmes AP, Rees G, Friston KJ 1998 Characterizing stimulus-response functions using nonlinear regressors in parametric fMRI experiments. Neuroimage. 8:140–148. 9740757 10.1006/nimg.1998.0351

[bhy322C19] Buckner RL 2013 The cerebellum and cognitive function: 25 years of insight from anatomy and neuroimaging. Neuron. 80:807–815. 24183029 10.1016/j.neuron.2013.10.044

[bhy322C20] Buckner RL, Krienen FM, Castellanos A, Diaz JC, Yeo BT 2011 The organization of the human cerebellum estimated by intrinsic functional connectivity. J Neurophysiol. 106:2322–2345. 21795627 10.1152/jn.00339.2011 PMC3214121

[bhy322C21] Caligiore D, Pezzulo G, Baldassarre G, Bostan AC, Strick PL, Doya K, Helmich RC, Dirkx M, Houk J, Jörntell H, et al 2017 Consensus paper: towards a systems-level view of cerebellar function: the interplay between cerebellum, basal ganglia, and cortex. Cerebellum. 16:203–229. 26873754 10.1007/s12311-016-0763-3 PMC5243918

[bhy322C22] Caligiore D, Pezzulo G, Miall RC, Baldassarre G 2013 The contribution of brain sub-cortical loops in the expression and acquisition of action understanding abilities. Neurosci Biobehav Rev. 37:2504–2515. 23911926 10.1016/j.neubiorev.2013.07.016 PMC3878436

[bhy322C23] Callan DE, Terzibas C, Cassel DB, Callan A, Kawato M, Sato MA 2013 Differential activation of brain regions involved with error-feedback and imitation based motor simulation when observing self and an expert’s actions in pilots and non-pilots on a complex glider landing task. Neuroimage. 72:55–68. 23357079 10.1016/j.neuroimage.2013.01.028

[bhy322C24] Calvo-Merino B, Grèzes J, Glaser DE, Passingham RE, Haggard P 2006 Seeing or doing? Influence of visual and motor familiarity in action observation. Curr Biol. 16:1905–1910. 17027486 10.1016/j.cub.2006.07.065

[bhy322C25] Campbell MEJ, Mehrkanoon S, Cunnington R 2018 Intentionally not imitating: Insula cortex engaged for top-down control of action mirroring. Neuropsychologia. 111:241–251. 29408525 10.1016/j.neuropsychologia.2018.01.037

[bhy322C26] Caspers S, Zilles K, Laird AR, Eickhoff SB 2010 ALE meta-analysis of action observation and imitation in the human brain. Neuroimage. 50:1148–1167. 20056149 10.1016/j.neuroimage.2009.12.112 PMC4981639

[bhy322C27] Cauda F, Costa T, Torta DM, Sacco K, D’Agata F, Duca S, Geminiani G, Fox PT, Vercelli A 2012 Meta-analytic clustering of the insular cortex: characterizing the meta-analytic connectivity of the insula when involved in active tasks. Neuroimage. 62:343–355. 22521480 10.1016/j.neuroimage.2012.04.012 PMC4782788

[bhy322C28] Cavallo A, Ansuini C, Becchio C 2015 The (un)coupling between action execution and observation: comment on “Grasping synergies: a motor-control approach to the mirror neuron mechanism” by D’Ausilio, Bartoli and Maffongelli. Phys Life Rev. 12:129–130. 25619152 10.1016/j.plrev.2015.01.006 PMC5040486

[bhy322C29] Cavanna AE, Trimble MR 2006 The precuneus: a review of its functional anatomy and behavioural correlates. Brain. 129:564–583. 16399806 10.1093/brain/awl004

[bhy322C30] Cheney PD, Fetz EE 1980 Functional classes of primate corticomotoneuronal cells and their relation to active force. J Neurophysiol. 44:773–791. 6253605 10.1152/jn.1980.44.4.773

[bhy322C31] Cheng K, Fujita H, Kanno I, Miura S, Tanaka K 1995 Human cortical regions activated by wide-field visual motion: an H2(15)O PET study. J Neurophysiol. 74:413–427. 7472342 10.1152/jn.1995.74.1.413

[bhy322C32] Christensen A, Giese MA, Sultan F, Mueller OM, Goericke SL, Ilg W, Timmann D 2014 An intact action-perception coupling depends on the integrity of the cerebellum. J Neurosci. 34:6707–6716. 24806697 10.1523/JNEUROSCI.3276-13.2014 PMC6608134

[bhy322C33] Cotterill RM 2001 Cooperation of the basal ganglia, cerebellum, sensory cerebrum and hippocampus: possible implications for cognition, consciousness, intelligence and creativity. Prog Neurobiol. 64:1–33. 11250060 10.1016/s0301-0082(00)00058-7

[bhy322C34] Craig AD 2003 Interoception: the sense of the physiological condition of the body. Curr Opin Neurobiol. 13:500–505. 12965300 10.1016/s0959-4388(03)00090-4

[bhy322C35] Cramer SC, Weisskoff RM, Schaechter JD, Nelles G, Foley M, Finklestein SP, Rosen BR 2002 Motor cortex activation is related to force of squeezing. Hum Brain Mapp. 16:197–205. 12112762 10.1002/hbm.10040 PMC6871791

[bhy322C36] Decety J, Grèzes J, Costes N, Perani D, Jeannerod M, Procyk E, Grassi F, Fazio F 1997 Brain activity during observation of actions. Influence of action content and subject’s strategy. Brain. 120(Pt 10):1763–1777. 9365369 10.1093/brain/120.10.1763

[bhy322C37] Di Cesare G, Valente G, Di Dio C, Ruffaldi E, Bergamasco M, Goebel R, Rizzolatti G 2016 Vitality forms processing in the insula during action observation: a multivoxel pattern analysis. Front Hum Neurosci. 10:267. 27375461 10.3389/fnhum.2016.00267 PMC4899476

[bhy322C38] Diedrichsen J 2006 A spatially unbiased atlas template of the human cerebellum. Neuroimage. 33:127–138. 16904911 10.1016/j.neuroimage.2006.05.056

[bhy322C39] Diedrichsen J, Balsters JH, Flavell J, Cussans E, Ramnani N 2009 A probabilistic MR atlas of the human cerebellum. Neuroimage. 46:39–46. 19457380 10.1016/j.neuroimage.2009.01.045

[bhy322C40] Diedrichsen J, White O, Newman D, Lally N 2010 Use-dependent and error-based learning of motor behaviors. J Neurosci. 30:5159–5166. 20392938 10.1523/JNEUROSCI.5406-09.2010 PMC6632748

[bhy322C41] Diedrichsen J, Zotow E 2015 Surface-based display of volume-averaged cerebellar imaging data. PLoS One. 10:e0133402. 26230510 10.1371/journal.pone.0133402 PMC4521932

[bhy322C42] Dupont P, Orban GA, De Bruyn B, Verbruggen A, Mortelmans L 1994 Many areas in the human brain respond to visual motion. J Neurophysiol. 72:1420–1424. 7807222 10.1152/jn.1994.72.3.1420

[bhy322C43] D’Angelo E, Casali S 2012 Seeking a unified framework for cerebellar function and dysfunction: from circuit operations to cognition. Front Neural Circuits. 6:116. 23335884 10.3389/fncir.2012.00116 PMC3541516

[bhy322C44] D’Angelo E, Gandini Wheeler-Kingshott C 2017 Modelling the brain: elementary components to explain ensemblefunctions. Rivista Del Nuovo Cimento. 40:36.

[bhy322C45] E KH, Chen SH, Ho MH, Desmond JE 2014 A meta-analysis of cerebellar contributions to higher cognition from PET and fMRI studies. Hum Brain Mapp. 35:593–615. 23125108 10.1002/hbm.22194 PMC3866223

[bhy322C46] Ebisch SJ, Perrucci MG, Ferretti A, Del Gratta C, Romani GL, Gallese V 2008 The sense of touch: embodied simulation in a visuotactile mirroring mechanism for observed animate or inanimate touch. J Cogn Neurosci. 20:1611–1623. 18345991 10.1162/jocn.2008.20111

[bhy322C47] Ehrsson HH, Fagergren E, Forssberg H 2001 Differential fronto-parietal activation depending on force used in a precision grip task: an fMRI study. J Neurophysiol. 85:2613–2623. 11387405 10.1152/jn.2001.85.6.2613

[bhy322C48] Evarts EV 1968 Relation of pyramidal tract activity to force exerted during voluntary movement. J Neurophysiol. 31:14–27. 4966614 10.1152/jn.1968.31.1.14

[bhy322C49] Fagg AH, Arbib MA 1998 Modeling parietal-premotor interactions in primate control of grasping. Neural Netw. 11:1277–1303. 12662750 10.1016/s0893-6080(98)00047-1

[bhy322C50] Filimon F, Nelson JD, Hagler DJ, Sereno MI 2007 Human cortical representations for reaching: mirror neurons for execution, observation, and imagery. Neuroimage. 37:1315–1328. 17689268 10.1016/j.neuroimage.2007.06.008 PMC2045689

[bhy322C51] Flandin G, Friston KJ 2017 Analysis of family-wise error rates in statistical parametric mapping using random field theory. Hum Brain Mapp. 10.1002/hbm.23839 PMC6585687 29091338

[bhy322C52] Friston K 2012 Ten ironic rules for non-statistical reviewers. Neuroimage. 61:1300–1310. 22521475 10.1016/j.neuroimage.2012.04.018

[bhy322C53] Friston KJ, Fletcher P, Josephs O, Holmes A, Rugg MD, Turner R 1998 Event-related fMRI: characterizing differential responses. Neuroimage. 7:30–40. 9500830 10.1006/nimg.1997.0306

[bhy322C54] Friston KJ, Mechelli A, Turner R, Price CJ 2000 Nonlinear responses in fMRI: the Balloon model, Volterra kernels, and other hemodynamics. Neuroimage. 12:466–477. 10988040 10.1006/nimg.2000.0630

[bhy322C55] Friston KJ, Williams S, Howard R, Frackowiak RS, Turner R 1996 Movement-related effects in fMRI time-series. Magn Reson Med. 35:346–355. 8699946 10.1002/mrm.1910350312

[bhy322C56] Garrison KA, Aziz-Zadeh L, Wong SW, Liew SL, Winstein CJ 2013 Modulating the motor system by action observation after stroke. Stroke. 44:2247–2253. 23743974 10.1161/STROKEAHA.113.001105 PMC3753677

[bhy322C57] Gatti R, Rocca MA, Fumagalli S, Cattrysse E, Kerckhofs E, Falini A, Filippi M 2017 The effect of action observation/execution on mirror neuron system recruitment: an fMRI study in healthy individuals. Brain Imaging Behav. 11:565–576. 27011016 10.1007/s11682-016-9536-3

[bhy322C58] Gazzola V, Keysers C 2009 The observation and execution of actions share motor and somatosensory voxels in all tested subjects: single-subject analyses of unsmoothed fMRI data. Cereb Cortex. 19:1239–1255. 19020203 10.1093/cercor/bhn181 PMC2677653

[bhy322C59] Glickstein M, Gerrits N, Kralj-Hans I, Mercier B, Stein J, Voogd J 1994 Visual pontocerebellar projections in the macaque. J Comp Neurol. 349:51–72. 7852626 10.1002/cne.903490105

[bhy322C60] Graybiel AM 1998 The basal ganglia and chunking of action repertoires. Neurobiol Learn Mem. 70:119–136. 9753592 10.1006/nlme.1998.3843

[bhy322C61] Grèzes J, Decety J 2001 Functional anatomy of execution, mental simulation, observation, and verb generation of actions: a meta-analysis. Hum Brain Mapp. 12:1–19. 11198101 10.1002/1097-0193(200101)12:1<1::AID-HBM10>3.0.CO;2-V PMC6872039

[bhy322C62] Hamilton AF, Grafton ST 2008 Action outcomes are represented in human inferior frontoparietal cortex. Cereb Cortex. 18:1160–1168. 17728264 10.1093/cercor/bhm150

[bhy322C63] Hermsdörfer J, Goldenberg G, Wachsmuth C, Conrad B, Ceballos-Baumann AO, Bartenstein P, Schwaiger M, Boecker H 2001 Cortical correlates of gesture processing: clues to the cerebral mechanisms underlying apraxia during the imitation of meaningless gestures. Neuroimage. 14:149–161. 11525324 10.1006/nimg.2001.0796

[bhy322C64] Ishibashi R, Pobric G, Saito S, Lambon Ralph MA 2016 The neural network for tool-related cognition: an activation likelihood estimation meta-analysis of 70 neuroimaging contrasts. Cogn Neuropsychol. 33:241–256. 27362967 10.1080/02643294.2016.1188798 PMC4989859

[bhy322C65] Ito M 2008 Control of mental activities by internal models in the cerebellum. Nat Rev Neurosci. 9:304–313. 18319727 10.1038/nrn2332

[bhy322C66] Jovicich J, Peters RJ, Koch C, Braun J, Chang L, Ernst T 2001 Brain areas specific for attentional load in a motion-tracking task. J Cogn Neurosci. 13:1048–1058. 11784443 10.1162/089892901753294347

[bhy322C67] Kasess CH, Windischberger C, Cunnington R, Lanzenberger R, Pezawas L, Moser E 2008 The suppressive influence of SMA on M1 in motor imagery revealed by fMRI and dynamic causal modeling. Neuroimage. 40:828–837. 18234512 10.1016/j.neuroimage.2007.11.040

[bhy322C68] Keisker B, Hepp-Reymond MC, Blickenstorfer A, Meyer M, Kollias SS 2009 Differential force scaling of fine-graded power grip force in the sensorimotor network. Hum Brain Mapp. 30:2453–2465. 19172654 10.1002/hbm.20676 PMC6871245

[bhy322C69] Kelly RM, Strick PL 2003 Cerebellar loops with motor cortex and prefrontal cortex of a nonhuman primate. J Neurosci. 23:8432–8444. 12968006 10.1523/JNEUROSCI.23-23-08432.2003 PMC6740694

[bhy322C70] Kilner JM 2011 More than one pathway to action understanding. Trends Cogn Sci. 15:352–357. 21775191 10.1016/j.tics.2011.06.005 PMC3389781

[bhy322C71] Kilner JM, Friston KJ, Frith CD 2007 Predictive coding: an account of the mirror neuron system. Cogn Process. 8:159–166. 17429704 10.1007/s10339-007-0170-2 PMC2649419

[bhy322C72] Kim SG, Ogawa S 2012 Biophysical and physiological origins of blood oxygenation level-dependent fMRI signals. J Cereb Blood Flow Metab. 32:1188–1206. 22395207 10.1038/jcbfm.2012.23 PMC3390806

[bhy322C73] Knauff M, Mulack T, Kassubek J, Salih HR, Greenlee MW 2002 Spatial imagery in deductive reasoning: a functional MRI study. Brain Res Cogn Brain Res. 13:203–212. 11958963 10.1016/s0926-6410(01)00116-1

[bhy322C74] Koul A, Cavallo A, Cauda F, Costa T, Diano M, Pontil M, Becchio C 2018 Action observation areas represent intentions from subtle kinematic features. Cereb Cortex. 28:2647–2654. 29722797 10.1093/cercor/bhy098 PMC5998953

[bhy322C75] Krekelberg B, Boynton GM, van Wezel RJ 2006 Adaptation: from single cells to BOLD signals. Trends Neurosci. 29:250–256. 16529826 10.1016/j.tins.2006.02.008

[bhy322C76] Kuhtz-Buschbeck JP, Ehrsson HH, Forssberg H 2001 Human brain activity in the control of fine static precision grip forces: an fMRI study. Eur J Neurosci. 14:382–390. 11553288 10.1046/j.0953-816x.2001.01639.x

[bhy322C77] Kuhtz-Buschbeck JP, Gilster R, Wolff S, Ulmer S, Siebner H, Jansen O 2008 Brain activity is similar during precision and power gripping with light force: an fMRI study. Neuroimage. 40:1469–1481. 18316207 10.1016/j.neuroimage.2008.01.037

[bhy322C78] Leech R, Braga R, Sharp DJ 2012 Echoes of the brain within the posterior cingulate cortex. J Neurosci. 32:215–222. 22219283 10.1523/JNEUROSCI.3689-11.2012 PMC6621313

[bhy322C79] Leung HC, Gore JC, Goldman-Rakic PS 2002 Sustained mnemonic response in the human middle frontal gyrus during on-line storage of spatial memoranda. J Cogn Neurosci. 14:659–671. 12126506 10.1162/08989290260045882

[bhy322C80] Llinás RR 2009 Inferior olive oscillation as the temporal basis for motricity and oscillatory reset as the basis for motor error correction. Neuroscience. 162:797–804. 19393291 10.1016/j.neuroscience.2009.04.045 PMC2861300

[bhy322C81] Luks TL, Simpson GV 2004 Preparatory deployment of attention to motion activates higher-order motion-processing brain regions. Neuroimage. 22:1515–1522. 15275908 10.1016/j.neuroimage.2004.04.008

[bhy322C82] McGregor HR, Gribble PL 2015 Changes in visual and sensory-motor resting-state functional connectivity support motor learning by observing. J Neurophysiol. 114:677–688. 25995349 10.1152/jn.00286.2015 PMC4512249

[bhy322C83] Mechelli A, Price CJ, Friston KJ 2001 Nonlinear coupling between evoked rCBF and BOLD signals: a simulation study of hemodynamic responses. Neuroimage. 14:862–872. 11554805 10.1006/nimg.2001.0876

[bhy322C84] Menon V, Adleman NE, White CD, Glover GH, Reiss AL 2001 Error-related brain activation during a Go/NoGo response inhibition task. Hum Brain Mapp. 12:131–143. 11170305 10.1002/1097-0193(200103)12:3<131::AID-HBM1010>3.0.CO;2-C PMC6872006

[bhy322C85] Miall RC 2003 Connecting mirror neurons and forward models. Neuroreport. 14:2135–2137. 14625435 10.1097/00001756-200312020-00001

[bhy322C86] Molenberghs P, Cunnington R, Mattingley JB 2012 Brain regions with mirror properties: a meta-analysis of 125 human fMRI studies. Neurosci Biobehav Rev. 36:341–349. 21782846 10.1016/j.neubiorev.2011.07.004

[bhy322C87] Molenberghs P, Johnson H, Henry JD, Mattingley JB 2016 Understanding the minds of others: a neuroimaging meta-analysis. Neurosci Biobehav Rev. 65:276–291. 27073047 10.1016/j.neubiorev.2016.03.020

[bhy322C88] Morin O, Grèzes J 2008 What is “mirror” in the premotor cortex? A review. Neurophysiol Clin. 38:189–195. 18539253 10.1016/j.neucli.2008.02.005

[bhy322C89] Munzert J, Lorey B, Zentgraf K 2009 Cognitive motor processes: the role of motor imagery in the study of motor representations. Brain Res Rev. 60:306–326. 19167426 10.1016/j.brainresrev.2008.12.024

[bhy322C90] Naish KR, Houston-Price C, Bremner AJ, Holmes NP 2014 Effects of action observation on corticospinal excitability: muscle specificity, direction, and timing of the mirror response. Neuropsychologia. 64:331–348. 25281883 10.1016/j.neuropsychologia.2014.09.034

[bhy322C91] Nakata H, Sakamoto K, Ferretti A, Gianni Perrucci M, Del Gratta C, Kakigi R, Luca Romani G 2008 Somato-motor inhibitory processing in humans: an event-related functional MRI study. Neuroimage. 39:1858–1866. 18083602 10.1016/j.neuroimage.2007.10.041

[bhy322C92] Neely KA, Coombes SA, Planetta PJ, Vaillancourt DE 2013 Segregated and overlapping neural circuits exist for the production of static and dynamic precision grip force. Hum Brain Mapp. 34:698–712. 22109998 10.1002/hbm.21467 PMC3292669

[bhy322C93] Oldfield RC 1971 The assessment and analysis of handedness: the Edinburgh inventory. Neuropsychologia. 9:97–113. 5146491 10.1016/0028-3932(71)90067-4

[bhy322C94] Palesi F, De Rinaldis A, Castellazzi G, Calamante F, Muhlert N, Chard D, Tournier JD, Magenes G, D’Angelo E, Gandini Wheeler-Kingshott CAM 2017 Contralateral cortico-ponto-cerebellar pathways reconstruction in humans in vivo: implications for reciprocal cerebro-cerebellar structural connectivity in motor and non-motor areas. Sci Rep. 7:12841. 28993670 10.1038/s41598-017-13079-8 PMC5634467

[bhy322C95] Palesi F, Tournier JD, Calamante F, Muhlert N, Castellazzi G, Chard D, D’Angelo E, Wheeler-Kingshott CA 2015 Contralateral cerebello-thalamo-cortical pathways with prominent involvement of associative areas in humans in vivo. Brain Struct Funct. 220:3369–3384. 25134682 10.1007/s00429-014-0861-2 PMC4575696

[bhy322C96] Pineda JA, Moore AR, Elfenbeinand H, Cox R 2009 Hierarchically organized mirroring processes in social cognition: the functional neuroanatomy of empathy In: Pineda JA, editor Mirror neuron systems: the role of mirroring processes in social cognition. Totowa, NJ: Humana Press p. 135–160.

[bhy322C97] Porro CA, Facchin P, Fusi S, Dri G, Fadiga L 2007 Enhancement of force after action observation: behavioural and neurophysiological studies. Neuropsychologia. 45:3114–3121. 17681358 10.1016/j.neuropsychologia.2007.06.016

[bhy322C98] Ptak R 2012 The frontoparietal attention network of the human brain: action, saliency, and a priority map of the environment. Neuroscientist. 18:502–515. 21636849 10.1177/1073858411409051

[bhy322C99] Ramnani N 2006 The primate cortico-cerebellar system: anatomy and function. Nat Rev Neurosci. 7:511–522. 16791141 10.1038/nrn1953

[bhy322C100] Reed CL, Shoham S, Halgren E 2004 Neural substrates of tactile object recognition: an fMRI study. Hum Brain Mapp. 21:236–246. 15038005 10.1002/hbm.10162 PMC6871926

[bhy322C101] Riehle A, MacKay WA, Requin J 1994 Are extent and force independent movement parameters? Preparation and movement-related neuronal activity in the monkey cortex. Exp Brain Res. 99:56–74. 7925796 10.1007/BF00241412

[bhy322C102] Rizzolatti G, Craighero L 2004 The mirror-neuron system. Annu Rev Neurosci. 27:169–192. 15217330 10.1146/annurev.neuro.27.070203.144230

[bhy322C103] Romaiguère P, Nazarian B, Roth M, Anton JL, Felician O 2014 Lateral occipitotemporal cortex and action representation. Neuropsychologia. 56:167–177. 24467888 10.1016/j.neuropsychologia.2014.01.006

[bhy322C104] Salama IM, Turner S, Edwards MG 2011 Automatic priming of grip force following action observation. Q J Exp Psychol (Hove). 64:833–838. 21512959 10.1080/17470218.2011.572172

[bhy322C105] Schmahmann JD 1996 From movement to thought: anatomic substrates of the cerebellar contribution to cognitive processing. Hum Brain Mapp. 4:174–198. 20408197 10.1002/(SICI)1097-0193(1996)4:3<174::AID-HBM3>3.0.CO;2-0

[bhy322C106] Schmahmann JD, Pandya DN 1995 Prefrontal cortex projections to the basilar pons in rhesus monkey: implications for the cerebellar contribution to higher function. Neurosci Lett. 199:175–178. 8577391 10.1016/0304-3940(95)12056-a

[bhy322C107] Schurz M, Radua J, Aichhorn M, Richlan F, Perner J 2014 Fractionating theory of mind: a meta-analysis of functional brain imaging studies. Neurosci Biobehav Rev. 42:9–34. 24486722 10.1016/j.neubiorev.2014.01.009

[bhy322C108] Sevdalis V, Keller PE 2011 Captured by motion: dance, action understanding, and social cognition. Brain Cogn. 77:231–236. 21880410 10.1016/j.bandc.2011.08.005

[bhy322C109] Shadmehr R, Mussa-Ivaldi S 2012 Biological learning and control. Cambridge, MA: The MIT Press..

[bhy322C110] Shima K, Tanji J 1998 Both supplementary and presupplementary motor areas are crucial for the temporal organization of multiple movements. J Neurophysiol. 80:3247–3260. 9862919 10.1152/jn.1998.80.6.3247

[bhy322C111] Shomstein S 2012 Cognitive functions of the posterior parietal cortex: top-down and bottom-up attentional control. Front Integr Neurosci. 6:38. 22783174 10.3389/fnint.2012.00038 PMC3389368

[bhy322C112] Smith AM, Hepp-Reymond MC, Wyss UR 1975 Relation of activity in precentral cortical neurons to force and rate of force change during isometric contractions of finger muscles. Exp Brain Res. 23:315–332. 810360 10.1007/BF00239743

[bhy322C113] Sokolov AA, Miall RC, Ivry RB 2017 The cerebellum: adaptive prediction for movement and cognition. Trends Cogn Sci. 21:313–332. 28385461 10.1016/j.tics.2017.02.005 PMC5477675

[bhy322C114] Spraker MB, Yu H, Corcos DM, Vaillancourt DE 2007 Role of individual basal ganglia nuclei in force amplitude generation. J Neurophysiol. 98:821–834. 17567775 10.1152/jn.00239.2007 PMC2367092

[bhy322C115] Stefan K, Cohen LG, Duque J, Mazzocchio R, Celnik P, Sawaki L, Ungerleider L, Classen J 2005 Formation of a motor memory by action observation. J Neurosci. 25:9339–9346. 16221842 10.1523/JNEUROSCI.2282-05.2005 PMC6725701

[bhy322C116] Stoodley CJ, Schmahmann JD 2010 Evidence for topographic organization in the cerebellum of motor control versus cognitive and affective processing. Cortex. 46:831–844. 20152963 10.1016/j.cortex.2009.11.008 PMC2873095

[bhy322C117] Stoodley CJ, Valera EM, Schmahmann JD 2012 Functional topography of the cerebellum for motor and cognitive tasks: an fMRI study. Neuroimage. 59:1560–1570. 21907811 10.1016/j.neuroimage.2011.08.065 PMC3230671

[bhy322C118] Strick PL, Dum RP, Fiez JA 2009 Cerebellum and nonmotor function. Annu Rev Neurosci. 32:413–434. 19555291 10.1146/annurev.neuro.31.060407.125606

[bhy322C119] Tanaka S, Inui T, Iwaki S, Konishi J, Nakai T 2001 Neural substrates involved in imitating finger configurations: an fMRI study. Neuroreport. 12:1171–1174. 11338186 10.1097/00001756-200105080-00024

[bhy322C120] Thompson JC, Hardee JE, Panayiotou A, Crewther D, Puce A 2007 Common and distinct brain activation to viewing dynamic sequences of face and hand movements. Neuroimage. 37:966–973. 17616403 10.1016/j.neuroimage.2007.05.058

[bhy322C121] Thompson J, Parasuraman R 2012 Attention, biological motion, and action recognition. Neuroimage. 59:4–13. 21640836 10.1016/j.neuroimage.2011.05.044

[bhy322C122] Turella L, Pierno AC, Tubaldi F, Castiello U 2009 Mirror neurons in humans: consisting or confounding evidence? Brain Lang. 108:10–21. 18082250 10.1016/j.bandl.2007.11.002

[bhy322C123] Valchev N, Zijdewind I, Keysers C, Gazzola V, Avenanti A, Maurits NM 2015 Weight dependent modulation of motor resonance induced by weight estimation during observation of partially occluded lifting actions. Neuropsychologia. 66:237–245. 25462196 10.1016/j.neuropsychologia.2014.11.030 PMC4966623

[bhy322C124] Van Overwalle F, Baetens K 2009 Understanding others’ actions and goals by mirror and mentalizing systems: a meta-analysis. Neuroimage. 48:564–584. 19524046 10.1016/j.neuroimage.2009.06.009

[bhy322C125] Van Overwalle F, Baetens K, Mariën P, Vandekerckhove M 2014 Social cognition and the cerebellum: a meta-analysis of over 350 fMRI studies. Neuroimage. 86:554–572. 24076206 10.1016/j.neuroimage.2013.09.033

[bhy322C126] Van Overwalle F, Baetens K, Mariën P, Vandekerckhove M 2015 Cerebellar areas dedicated to social cognition? A comparison of meta-analytic and connectivity results. Soc Neurosci. 10:337–344. 25621820 10.1080/17470919.2015.1005666

[bhy322C127] Van Overwalle F, D’aes T, Mariën P 2015 Social cognition and the cerebellum: a meta-analytic connectivity analysis. Hum Brain Mapp. 36:5137–5154. 26419890 10.1002/hbm.23002 PMC6869534

[bhy322C128] Van Overwalle F, Mariën P 2016 Functional connectivity between the cerebrum and cerebellum in social cognition: a multi-study analysis. Neuroimage. 124:248–255. 26348560 10.1016/j.neuroimage.2015.09.001

[bhy322C129] Vanderwert RE, Fox NA, Ferrari PF 2013 The mirror mechanism and mu rhythm in social development. Neurosci Lett. 540:15–20. 23063953 10.1016/j.neulet.2012.10.006 PMC3612380

[bhy322C130] Ward NS, Frackowiak RS 2003 Age-related changes in the neural correlates of motor performance. Brain. 126:873–888. 12615645 10.1093/brain/awg071 PMC3717766

[bhy322C131] Wasson P, Prodoehl J, Coombes SA, Corcos DM, Vaillancourt DE 2010 Predicting grip force amplitude involves circuits in the anterior basal ganglia. Neuroimage. 49:3230–3238. 19944767 10.1016/j.neuroimage.2009.11.047 PMC2818558

[bhy322C132] Watson TC, Becker N, Apps R, Jones MW 2014 Back to front: cerebellar connections and interactions with the prefrontal cortex. Front Syst Neurosci. 8:4. 24550789 10.3389/fnsys.2014.00004 PMC3912388

[bhy322C133] Weaver J 2015 Motor learning unfolds over different timescales in distinct neural systems. PLoS Biol. 13:e1002313. 26646076 10.1371/journal.pbio.1002313 PMC4672876

[bhy322C134] Weigelt S, Muckli L, Kohler A 2008 Functional magnetic resonance adaptation in visual neuroscience. Rev Neurosci. 19:363–380. 19145990 10.1515/revneuro.2008.19.4-5.363

[bhy322C135] Wiggett AJ, Downing PE 2011 Representation of action in occipito-temporal cortex. J Cogn Neurosci. 23:1765–1780. 20807060 10.1162/jocn.2010.21552

[bhy322C136] Yamazaki T, Nagao S 2012 A computational mechanism for unified gain and timing control in the cerebellum. PLoS One. 7:e33319. 22438912 10.1371/journal.pone.0033319 PMC3305129

[bhy322C137] Yeo BT, Krienen FM, Sepulcre J, Sabuncu MR, Lashkari D, Hollinshead M, Roffman JL, Smoller JW, Zöllei L, Polimeni JR, et al 2011 The organization of the human cerebral cortex estimated by intrinsic functional connectivity. J Neurophysiol. 106:1125–1165. 21653723 10.1152/jn.00338.2011 PMC3174820

