# Supplementary material for: I See Your Effort: Force-Related BOLD Effects in an Extended Action Execution–Observation Network Involving the Cerebellum
Source: Cereb Cortex. 2019 Jan 7;29(3):1351–68. doi: 10.1093/cercor/bhy322 (PMC6373696; doi:10.1093/cercor/bhy322)
Supplement: Supplementary Data [file bhy322_casiraghi2018cc_sm.docx]

**SUPPLEMENTARY MATERIAL**

**fMRI RESULTS**

Specific fMRI results on AE, AO and conjunction analysis are here reported with tables detailing significant effects (including coordinates, T values and cluster extent).

***Action execution***

Regionally specific effects in the AE condition (*Tables 1sm and 2sm*) were detected as follows (*p<0.05 FWE corr*); 0^th^ order: precentral and postcentral gyri (contralateral to the hand used in the task), cerebellum (lobules V, VI and VIII), occipital gyri, parietal lobules, cingulate cortex; +1^st^ order: precentral gyrus, hippocampus*,* basal forebrain, cerebellum (lobules V, VI and vermis VI), superior parietal lobule*,* calcarine gyri; -1^st^ order: cerebellar lobule IX; +2^nd^ order: precentral and postcentral gyri, putamen, insular lobe, parietal lobules, anterior cingulate gyri*,* medial and superior frontal gyri, occipital gyrus, fusiform gyrus, rolandic operculum, and cerebellum (lobule VI); -3^rd^ order: occipital and fusiform gyri*,* precentral and postcentral gyri, superior and inferior frontal gyrus (pars opercularis), supramarginal gyrus, putamen, insula, caudate, and pallidum; +4^th^ order: precentral and postcentral gyri, occipital, lingual and fusiform gyri, middle and posterior cingulate cortex, and superior parietal lobule. Among other regions, cerebellar effects were also found for -3^rd^ order but did not survive the correction for multiple comparisons. No effects were found for the +3^rd^, -2^nd^ and -4^th^ orders. Overall, thresholding at p<0.05, FWE regionally specific effects were therefore found in the 0^th^, +1^st^, +2^nd^, -1^st^ and -3^rd^ orders only.

**Table 1sm| Brain regions that are activated for action execution.** This table shows the main effect of hand gripping (0^th^ order) and force-related effects (1^st^, 2^nd^, 3^rd^ and 4^th^ orders) on BOLD signal reported for the five polynomial coefficients that resulted in significant clusters. The statistical threshold is set using p_u_<0.001 (k≥10) at the voxel level.

*Clusters in italic have a minor extension (k<10)*. Regions (*) are corrected (p<0.05) for multiple comparisons at the cluster level. The last column reports the probability (%) of these voxels being located in the respective Brodmann area (BA) or specific location (Loc) according to the cytoarchitectonic maps. ext = number of voxels in a cluster; T = T-value at the voxel level. x, y, z are peak coordinates in MNI space (mm).

| **Cluster** | | **Peak** | | | | | |
| --- | --- | --- | --- | --- | --- | --- | --- |
| **p_u_** | **ext** | **T** | **x** | **y** | **z** | **Anatomical region** | **BA/Loc (%)** |
| **0^th^ order** | | | | | | | |
| k≥10 | | | | | | | |
| 0.000 | 11267 | 18.36 | 39 | -67 | -11 | R Inferior Occipital Gyrus * | FG1 (50) |
|  |  | 17.7 | -39 | -31 | 61 | L Postcentral Gyrus * | 4a (44) |
|  |  | 16.8 | -36 | -67 | -8 | L Inferior Occipital Gyrus * | FG2 (21) |
|  |  | 16.12 | -36 | -28 | 52 | L Postcentral Gyrus * | 4p (49) |
|  |  | 15.46 | -45 | -25 | 58 | L Postcentral Gyrus * | 1 (51) |
|  |  | 15.03 | -42 | -73 | -14 | L Inferior Occipital Gyrus * | FG2 (46) |
|  |  | 14.84 | 18 | -55 | -23 | R Cerebellum (VI) * | Lobule VI (Hem) (90) |
|  |  | 14.8 | -39 | -22 | 55 | L Postcentral Gyrus * | 4a (54) |
|  |  | 14.65 | -42 | -19 | 58 | L Precentral Gyrus * | 4a (12) |
|  |  | 14.02 | -27 | -55 | -20 | L Cerebellum (VI) * | Lobule VI (Hem) (72) |
|  |  | 13.96 | 33 | -64 | -20 | R Cerebellum (VI) * | FG1 (56) |
|  |  | 13.49 | 48 | -76 | 1 | R Middle Occipital Gyrus * | hOc4la (87) |
|  |  | 13.39 | -36 | -79 | -8 | L Inferior Occipital Gyrus * | hOc4la (48) |
|  |  | 13.3 | 0 | -1 | 46 | L Middle Cingulate Cortex * |  |
|  |  | 13.28 | 54 | -28 | 40 | R Supramarginal Gyrus * | PFt (IPL) (61) |
| k<10 | | | | | | | |
| *0.264* | *5* | *4.89* | *27* | *-22* | *-8* | *Thal: Temporal* | *Thal: Temporal (48)* |
| **1^st^ order** | | | | | | | |
| k≥10 | | | | | | | |
| 0.000 | 1046 | 15.31 | -30 | -28 | 55 | L Precentral Gyrus * | 4p (47) |
|  |  | 12.43 | -21 | -25 | 67 | L Precentral Gyrus * | 4a (20) |
|  |  | 10.04 | -36 | -22 | 49 | L Postcentral Gyrus * | 4p (73) |
| 0.000 | 157 | 6.83 | -15 | -16 | 25 | Thal: Temporal * | Thal: Temporal (17) |
| 0.007 | 49 | 4.73 | -36 | -40 | -5 | CA1 (Hippocampus) * | CA1 (Hippocampus) (5) |
| 0.000 | 527 | 7.84 | -9 | -10 | 52 | L Posterior-Medial Frontal * |  |
| 0.000 | 477 | 7.24 | 6 | -61 | -8 | Lobule V (Hem) * | Lobule V (Hem) (49) |
|  |  | 6.41 | 18 | -52 | -20 | Lobule VI (Hem) * | Lobule VI (Hem) (80) |
|  |  | 6.21 | 6 | -64 | -23 | Cerebellar Vermis (6) * | Lobule VI (Verm) (47) |
| 0.014 | 38 | 7.13 | -15 | -1 | -11 | Basal Forebrain (Ch 4) * | BF (Ch 4) (19) |
| 0.017 | 36 | 6.82 | 15 | -88 | 22 | R Cuneus | hOc4d [V3A] (55) |
| 0.000 | 115 | 6.8 | 18 | -46 | 55 | Area 5L (SPL) * | 5L (SPL) (30) |
|  |  | 4.13 | 18 | -61 | 49 | Area 7A (SPL) * | 7A (SPL) (8) |
| 0.084 | 17 | 6.1 | -27 | 47 | 47 | L Middle Orbital Gyrus |  |
| 0.084 | 17 | 5.92 | 24 | 50 | 50 | R Superior Frontal Gyrus | Fp1 (72) |
| 0.032 | 28 | 5.91 | 21 | -25 | -14 | R Parahippocampal Gyrus | Subiculum (55) |
| 0.254 | 7 | 5.89 | 24 | -76 | 4 | Area hOc1 [V1] | hOc1 [V1] (36) |
| 0.001 | 82 | 5.45 | 6 | -85 | 1 | R Calcarine Gyrus * | hOc1 [V1] (79) |
|  |  | 5.15 | -6 | -94 | 13 | L Calcarine Gyrus * | hOc2 [V2] (38) |
|  |  | 4.94 | -3 | -88 | 22 | L Cuneus | hOc3d [V3d] (41) |
| 0.114 | 14 | 5.05 | 27 | -22 | 64 | R Precentral Gyrus |  |
| 0.157 | 11 | 5 | -42 | -79 | 7 | L Middle Occipital Gyrus | hOc4la (55) |
| 0.157 | 11 | 4.46 | 39 | -25 | 52 | R Postcentral Gyrus | 4p (60) |
| k<10 | | | | | | | |
| *0.254* | *7* | *4.62* | *0* | *-37* | *52* | *L Middle Cingulate Cortex* | *5M (SPL) (28)* |
| *0.290* | *6* | *4.49* | *-21* | *2* | *-20* | *L Amygdala* | *Amygdala (LB) (36)* |
| *0.290* | *6* | *4.44* | *54* | *-25* | *16* | *R Superior Temporal Gyrus* | *OP1 [SII] (52)* |
| **-1^st^ order** | | | | | | | |
| k≥10 | | | | | | | |
| 0.02 | 34 | 12.01 | 6 | -49 | -35 | R Cerebellum IX * | Lobule IX (Hem) (59) |
| **2^nd^ order** | | | | | | | |
| k≥10 | | | | | | | |
| 0.000 | 129 | 9.6 | -21 | 5 | -8 | L Putamen * |  |
|  |  | 6.81 | -33 | 17 | -2 | L Insular Lobe * |  |
| 0.000 | 705 | 8.63 | 48 | -31 | 34 | Area PFt  (Inferior Parietal Lobule) * | PFt (11) |
|  |  | 8.17 | 51 | -25 | 43 | R Postcentral Gyrus * | 2 (58) |
|  |  | 6.94 | 54 | -10 | 43 | R Precentral Gyrus * | 3b (10) |
| 0.000 | 385 | 8.55 | 9 | 29 | 22 | R Anterior Cingulate Cortex * | 33 (1) |
|  |  | 7.18 | -3 | -16 | 55 | L Posterior-Medial Frontal * |  |
|  |  | 6.69 | 0 | 8 | 28 | L Anterior Cingulate Cortex * |  |
| 0.000 | 764 | 7.19 | -33 | -37 | 37 | Area hIP1 (IPS) * | hIP1 (IPS) (4) |
|  |  | 6.93 | -51 | -34 | 31 | L Supramarginal Gyrus * | PFt (IPL) (37) |
|  |  | 6.81 | -57 | 8 | 22 | L Precentral Gyrus * | 44 (61) |
| 0.000 | 498 | 7.18 | -15 | -100 | 4 | L Middle Occipital Gyrus * | hOc3d [V3d] (41) |
|  |  | 6.54 | -39 | -70 | 1 | L Middle Occipital Gyrus * | hOc5 [V5/MT] (13) |
|  |  | 6.41 | -33 | -61 | -11 | L Fusiform Gyrus * | FG3 (35) |
| 0.000 | 341 | 7.05 | 33 | -70 | -14 | R Fusiform Gyrus * | FG1 (50) |
|  |  | 6.34 | 21 | -64 | -20 | R Cerebellum (VI) * | Lobule VI (Hem) (95) |
| 0.001 | 81 | 6.86 | 39 | -85 | 7 | R Middle Occipital Gyrus * | hOc4lp (76) |
| 0.001 | 65 | 6.68 | 33 | 8 | -2 | R Putamen * |  |
|  |  | 4.49 | 24 | 8 | -17 | R Olfactory Cortex * |  |
| 0.003 | 57 | 6.1 | -39 | -1 | 16 | L Rolandic Operculum * |  |
|  |  | 5.16 | -36 | -1 | 7 | L Insular Lobe * |  |
| 0.029 | 26 | 6.08 | -21 | -7 | 52 | L Superior Frontal Gyrus * |  |
| 0.002 | 61 | 5.87 | 39 | -13 | 58 | R Precentral Gyrus * |  |
|  |  | 4.83 | 45 | -28 | 61 | R Postcentral Gyrus * | 1 (76) |
|  |  | 4.67 | 39 | -1 | 58 | R Middle Frontal Gyrus * |  |
| 0.001 | 77 | 5.71 | 60 | 8 | 28 | R Precentral Gyrus * | 44 (25) |
|  |  | 5.01 | 57 | 14 | 19 | R Inferior Frontal Gyrus  (p. Opercularis) * | 44 (63) |
| 0.027 | 27 | 5.59 | -33 | 38 | 16 | L Middle Frontal Gyrus |  |
|  |  | 5.19 | -33 | 32 | 22 | L Middle Frontal Gyrus |  |
| 0.039 | 23 | 5.46 | -48 | 17 | -2 | L Inferior Frontal Gyrus  (p. Triangularis) | 44 (37) |
| 0.070 | 17 | 4.72 | 36 | 20 | 4 | R Insular Lobe | 45 (9) |
| k<10 | | | | | | | |
| *0.265* | *6* | *5.26* | *45* | *-64* | *-32* | *R Cerebellum (Crus 1)* | *Lobule VIIa crusI (Hem)* |
| *0.265* | *6* | *4.89* | *51* | *26* | *25* | *R Inferior Frontal Gyrus*  *(p. Triangularis)* | *45 (23)* |
| *0.265* | *6* | *4.67* | *24* | *-1* | *55* | *R Superior Frontal Gyrus* |  |
| *0.229* | *7* | *4.62* | *-18* | *5* | *61* | *L Superior Frontal Gyrus* |  |
| *0.308* | *5* | *4.43* | *-15* | *-73* | *-47* | *L Cerebellum (VII)* | *Lobule VIIb (Hem) (75)* |
| *0.265* | *6* | *4.41* | *30* | *50* | *28* | *R Middle Frontal Gyrus* |  |
| *0.229* | *7* | *4.4* | *-6* | *23* | *31* | *L Anterior Cingulate Cortex* |  |
| *0.308* | *5* | *4.4* | *36* | *41* | *16* | *R Middle Frontal Gyrus* |  |
| *0.265* | *6* | *4.33* | *9* | *-76* | *-44* | *R Cerebellum (VII)* | *Lobule VIIb (Hem) (47)* |
| **-3^rd^ order** | | | | | | | |
| k≥10 | | | | | | | |
| 0.000 | 1611 | 12.46 | 30 | -73 | -14 | R Fusiform Gyrus * | hOc4v [V4(v)] (73) |
|  |  | 11.04 | 36 | -82 | 4 | R Middle Occipital Gyrus * | hOc4lp (58) |
|  |  | 10.9 | 27 | -67 | 34 | R Superior Occipital Gyrus * |  |
| 0.000 | 2825 | 11.96 | -30 | -85 | 16 | L Middle Occipital Gyrus * | hOc4lp (28) |
|  |  | 11.14 | -21 | -73 | 31 | L Superior Occipital Gyrus * |  |
|  |  | 10.98 | -30 | -34 | 58 | L Postcentral Gyrus * | 4p (54) |
| 0.000 | 74 | 11.27 | -57 | 8 | 31 | L Precentral Gyrus * | 44 (45) |
| 0.000 | 335 | 8.86 | -24 | 2 | -8 | L Putamen * |  |
|  |  | 6.5 | -27 | 20 | 13 | L Insular Lobe * |  |
|  |  | 6.33 | -18 | -1 | 19 | L Caudate Nucleus * |  |
| 0.000 | 125 | 8.17 | 15 | 17 | -5 | R Caudate Nucleus * |  |
|  |  | 6.31 | 24 | 2 | -2 | R Pallidum * |  |
|  |  | 6.12 | 18 | 2 | -11 | Basal Forebrain (Ch 4) * | BF (Ch 4) (22) |
| 0.000 | 133 | 7.87 | 57 | 8 | 31 | R Precentral Gyrus * | 44 (29) |
|  |  | 5.53 | 48 | 2 | 28 | R Precentral Gyrus * | 44 (4) |
|  |  | 5.48 | 57 | 8 | 13 | R Inferior Frontal Gyrus  (p. Opercularis) * | 44 (51) |
| 0.022 | 26 | 7.04 | -51 | -16 | 22 | L Postcentral Gyrus |  |
| 0.000 | 180 | 6.89 | 39 | -13 | 58 | R Precentral Gyrus * |  |
|  |  | 6.59 | 24 | -7 | 61 | R Superior Frontal Gyrus * |  |
|  |  | 5.72 | 24 | -4 | 52 | R Superior Frontal Gyrus * |  |
| 0.000 | 103 | 6.88 | 57 | -16 | 28 | R Supramarginal Gyrus * | PFop (IPL) (29) |
|  |  | 5.06 | 57 | -19 | 13 | R Rolandic Operculum * | OP1 [SII] (54) |
|  |  | 4.86 | 63 | -13 | 16 | R Postcentral Gyrus * | OP4 [PV] (50) |
| 0.016 | 29 | 4.83 | 9 | -25 | 1 | R Thalamus | Thal: Prefrontal (51) |
| 0.006 | 40 | 6.41 | -45 | -31 | 19 | L Superior Temporal Gyrus * | OP1 [SII] (33) |
| 0.101 | 12 | 6.02 | 33 | -46 | -41 | Lobule VI (Hem) | Lobule VI (Hem) (26) |
| 0.115 | 11 | 5.57 | 33 | -28 | 40 | Area 3a | 3a (27) |
| 0.062 | 16 | 5.48 | -6 | -40 | 7 | L Posterior Cingulate Cortex |  |
| 0.050 | 18 | 5.12 | -15 | -22 | 7 | L Thalamus | Thal: Prefrontal (48) |
| 0.029 | 23 | 5.01 | -24 | 2 | 64 | L Superior Frontal Gyrus |  |
|  |  | 4.3 | -18 | -1 | 55 | L Superior Frontal Gyrus |  |
|  |  | 4.15 | -18 | -7 | 64 | L Posterior-Medial Frontal |  |
| 0.115 | 11 | 5.01 | -24 | 44 | 34 | L Middle Frontal Gyrus |  |
|  |  | 4.36 | -30 | 47 | 28 | L Middle Frontal Gyrus |  |
| 0.132 | 10 | 4.62 | 27 | 50 | 37 | R Middle Frontal Gyrus |  |
|  |  | 4.11 | 33 | 41 | 40 | R Middle Frontal Gyrus |  |
| 0.115 | 11 | 4.46 | -33 | 35 | 40 | L Middle Frontal Gyrus |  |
|  |  | 4.44 | -33 | 29 | 34 | L Middle Frontal Gyrus |  |
| k<10 | | | | | | | |
| *0.236* | *6* | *4.9* | *27* | *29* | *31* | *R Middle Frontal Gyrus* |  |
| *0.174* | *8* | *4.85* | *42* | *26* | *34* | *R Middle Frontal Gyrus* |  |
| *0.202* | *7* | *4.56* | *3* | *-46* | *49* | *R Precuneus* | *5M (SPL) (27)* |
| *0.202* | *7* | *5.76* | *-30* | *-10* | *4* | *L Putamen* |  |
| *0.202* | *7* | *6.07* | *-27* | *44* | *13* | *L Middle Frontal Gyrus* |  |
| *0.279* | *5* | *5.4* | *36* | *32* | *49* | *R Middle Frontal Gyrus* |  |
| *0.236* | *6* | *4.32* | *-30* | *56* | *19* | *L Middle Frontal Gyrus* | *Fp1 (3)* |
| **4^th^ order** | | | | | | | |
| k≥10 | | | | | | | |
| 0.000 | 481 | 8.36 | -27 | -31 | 61 | L Postcentral Gyrus * | 4p (37) |
|  |  | 7.01 | -33 | -16 | 52 | L Precentral Gyrus * |  |
|  |  | 6.76 | -54 | -16 | 34 | L Postcentral Gyrus * | 3b (81) |
| 0.000 | 612 | 7.72 | 24 | -79 | 16 | R Superior Occipital Gyrus * |  |
|  |  | 6.63 | 30 | -55 | -14 | R Fusiform Gyrus * | FG3 (68) |
|  |  | 6.47 | 30 | -70 | -8 | R Fusiform Gyrus * | hOc4v [V4(v)] (41) |
| 0.000 | 302 | 7.27 | -6 | -43 | 16 | L Posterior Cingulate Cortex * |  |
|  |  | 6.21 | 15 | -16 | 43 | R Middle Cingulate Cortex * |  |
|  |  | 6.09 | -6 | -22 | 49 | L Middle Cingulate Cortex * | 4a (8) |
| 0.002 | 73 | 6.8 | -57 | 5 | 28 | L Precentral Gyrus * | 44 (36) |
|  |  | 4.86 | -48 | -1 | 28 | L Precentral Gyrus * | 44 (15) |
| 0.014 | 40 | 6.32 | -21 | -7 | 7 | L Pallidum | Thal: Premotor (8) |
|  |  | 4.94 | -27 | 5 | -8 | L Putamen |  |
| 0.000 | 515 | 6.3 | -30 | -91 | 10 | L Middle Occipital Gyrus * | hOc4lp (73) |
|  |  | 5.98 | -39 | -76 | 4 | L Middle Occipital Gyrus * | hOc5 [V5/MT] (23) |
|  |  | 5.98 | -36 | -79 | 13 | L Middle Occipital Gyrus * | hOc4la (32) |
| 0.002 | 69 | 6.26 | 36 | -37 | 52 | R Postcentral Gyrus * | 2 (66) |
|  |  | 4.88 | 27 | -31 | 64 | R Postcentral Gyrus * | 4a (31) |
| 0.056 | 22 | 6.09 | -36 | -4 | 16 | L Insular Lobe |  |
| 0.002 | 74 | 6.07 | 3 | -61 | 4 | R Lingual Gyrus * | hOc1 [V1] (42) |
|  |  | 4.86 | -9 | -52 | -2 | L Lingual Gyrus * | hOc2 [V2] (21) |
|  |  | 4.81 | -9 | -61 | 4 | L Calcarine Gyrus * | hOc1 [V1] (51) |
| 0.044 | 25 | 5.9 | -3 | -16 | -5 | Thal: Prefrontal | Thal: Prefrontal (21) |
| 0.000 | 147 | 5.66 | -21 | -73 | 34 | L Superior Occipital Gyrus * |  |
|  |  | 5.54 | -18 | -67 | 40 | L Superior Parietal Lobule * |  |
|  |  | 5.44 | -18 | -79 | 40 | L Superior Occipital Gyrus * | hOc4d [V3A] (13) |
| 0.067 | 20 | 5.61 | -18 | -46 | -23 | L Cerebellum (IV-V) | Lobule V (Hem) (68) |
| 0.002 | 73 | 5.57 | 45 | -1 | 19 | Area 44 * | 44 (11) |
|  |  | 5.42 | 51 | 2 | 28 | R Precentral Gyrus * | 44 (2) |
|  |  | 4.93 | 54 | -4 | 43 | R Precentral Gyrus * | Area 3b (9) |
| 0.037 | 27 | 5.24 | 9 | -79 | -38 | R Cerebellum (Crus 2) | Lobule VIIa crusII (Hem) (45) |
| 0.074 | 19 | 5.18 | 27 | -52 | 58 | R Superior Parietal Lobule | 7PC (SPL) (55) |
| 0.034 | 28 | 5.09 | 51 | -16 | 31 | R Postcentral Gyrus | 3b (39) |
| 0.183 | 10 | 5.03 | 0 | -49 | 43 | L Precuneus |  |
| 0.021 | 34 | 4.86 | 33 | 5 | -2 | R Putamen |  |
|  |  | 4.49 | 24 | 8 | -2 | R Putamen |  |
| 0.108 | 15 | 4.82 | 9 | -1 | 55 | R Posterior-Medial Frontal |  |
| 0.098 | 16 | 4.81 | -39 | -46 | -14 | L Inferior Temporal Gyrus | FG4 (73) |
|  |  | 4.22 | -36 | -49 | -23 | L Fusiform Gyrus | FG4 (47) |
| 0.040 | 26 | 4.58 | 36 | -10 | 55 | R Precentral Gyrus |  |
| k<10 | | | | | | | |
| *0.262* | *7* | *4.7* | *9* | *-52* | *-11* | *R Cerebellum (IV-V)* | *Lobule V (Hem) (95)* |
| *0.205* | *9* | *4.6* | *15* | *-43* | *55* | *Area 3b* | *3b (5)* |
| *0.262* | *7* | *4.41* | *-21* | *-61* | *55* | *L Superior Parietal Lobule* | *7A (SPL) (45)* |

**Table 2sm| Brain regions that are activated for action execution.** This table shows the main effect of hand gripping (0^th^ order) and force related effects (1^st^, 2^nd^, 3^rd^ and 4^th^ orders) on BOLD signal reported for the five polynomial coefficients that resulted in significant clusters. Regions are voxel-wise corrected p<0.05 (FWE), k≥10. *Clusters in italic have a minor extension (k<10)*. The last column reports the probability (%) of these voxels being located in the respective Brodmann area (BA) or specific location (Loc) according to the cytoarchitectonic maps. p (FWE corr) = p-value corrected at the cluster level; ext = number of voxels in a cluster; T = T-value at the voxel level. x, y, z are peak coordinates in MNI space (mm).

| **Cluster** | | **Peak** | | | | | |
| --- | --- | --- | --- | --- | --- | --- | --- |
| **p (FWE corr)** | **ext** | **T** | **x** | **y** | **z** | **Anatomical region** | **BA/Loc (%)** |
| **0^th^ order** | | | | | | | |
| k≥10 | | | | | | | |
| 0.000 | 301 | 18.36 | 39 | -67 | -11 | R Inferior Occipital Gyrus | FG1 (49) |
|  |  | 14.84 | 18 | -55 | -23 | R Cerebellum (VI) | Lobule VI (Hem) (89) |
|  |  | 13.96 | 33 | -64 | -20 | R Cerebellum (VI) |  |
| 0.000 | 168 | 17.7 | -39 | -31 | 61 | L Postcentral Gyrus | 4a (44) |
|  |  | 16.12 | -36 | -28 | 52 | L Postcentral Gyrus | 4p (49) |
|  |  | 15.46 | -45 | -25 | 58 | L Postcentral Gyrus | 1 (50) |
| 0.000 | 151 | 16.8 | -36 | -67 | -8 | L Inferior Occipital Gyrus |  |
|  |  | 15.03 | -42 | -73 | -14 | L Inferior Occipital Gyrus | FG2 (46) |
|  |  | 13.39 | -36 | -79 | -8 | L Inferior Occipital Gyrus | hOc4la |
| 0.000 | 77 | 14.02 | -27 | -55 | -20 | L Cerebellum (VI) | Lobule VI (Hem) (72) |
|  |  | 10.2 | -18 | -70 | -17 | L Cerebellum (VI) | Lobule VI (Hem) (100) |
| 0.000 | 25 | 13.3 | 0 | -1 | 46 | L Middle Cingulate Cortex |  |
| 0.000 | 22 | 13.28 | 54 | -28 | 40 | R Supramarginal Gyrus | PFt (IPL) (61) |
|  |  | 13.08 | 57 | -16 | 31 | R Postcentral Gyrus | 3b (29) |
| 0.000 | 22 | 13.27 | -24 | -19 | 64 | L Precentral Gyrus |  |
| 0.000 | 21 | 12.53 | -33 | -46 | 58 | L Superior Parietal Lobule | 7PC (SPL) (50) |
| 0.000 | 27 | 12.08 | 15 | -70 | -50 | R Cerebellum (VIII) | Lobule VIIIa (Hem) (74) |
| 0.000 | 12 | 11.96 | 33 | -10 | 55 | R Precentral Gyrus |  |
| 0.000 | 10 | 10.59 | 3 | -64 | -11 | Cerebellar Vermis (6) | Lobule V (Hem) (80) |
|  | 10 | 9.62 | -60 | -22 | 40 | L Supramarginal Gyrus | PFt (IPL) (41) |
| k<10 | | | | | | | |
| 0.000 | 9 | 12.65 | 21 | -13 | 70 | R Superior Frontal Gyrus |  |
| 0.000 | 5 | 11.65 | -39 | -1 | 16 | L Rolandic Operculum |  |
| 0.000 | 7 | 11.36 | 24 | -61 | 61 | R Superior Parietal Lobule | 7A (SPL) (73) |
| 0.000 | 6 | 11.26 | -18 | -4 | 70 | L Superior Frontal Gyrus |  |
| 0.000 | 5 | 11.07 | -24 | -58 | 58 | L Superior Parietal Lobule | 7A (SPL) (55) |
| 0.000 | 9 | 10.91 | -54 | -19 | 34 | L Postcentral Gyrus | 3b (41) |
| 0.000 | 5 | 10.77 | 30 | -49 | 55 | R Inferior Parietal Lobule | 7PC (SPL) (49) |
| 0.001 | 3 | 10.71 | -6 | -19 | 46 | L Middle Cingulate Cortex |  |
| 0.000 | 5 | 10.7 | 30 | -40 | 49 | Area 2 | 2 (70) |
| 0.000 | 4 | 10.2 | 54 | 5 | 46 | R Precentral Gyrus |  |
| 0.000 | 7 | 10.07 | 42 | -34 | 52 | R Postcentral Gyrus | 2 (65) |
| 0.001 | 3 | 9.35 | 27 | -52 | -50 | R Cerebellum (VIII) | Lobule VIIIa (Hem) (47) |
| **1^st^ order** | | | | | | | |
| k≥10 | | | | | | | |
| 0.000 | 40 | 15.31 | -30 | -28 | 55 | L Precentral Gyrus | 4p (47) |
|  |  | 12.43 | -21 | -25 | 67 | L Precentral Gyrus | 4a (20) |
|  |  | 10.04 | -36 | -22 | 49 | L Postcentral Gyrus | 4p (73) |
| **-1^st^ order** | | | | | | | |
| k<10 | | | | | | | |
| 0.002 | 3 | 12.01 | 6 | -49 | -35 | R Cerebellum (IX) | Lobule IX (59) |
| **2^nd^ order** | | | | | | | |
| k<10 | | | | | | | |
| 0.001 | 3 | 9.6 | -21 | 5 | -8 | L Putamen |  |
| **-3^rd^ order** | | | | | | | |
| k≥10 | | | | | | | |
| 0.000 | 43 | 12.46 | 30 | -73 | -14 | R Fusiform Gyrus | hOc4v [V4(v)] (72) |
|  |  | 11.04 | 36 | -82 | 4 | R Middle Occipital Gyrus | hOc4lp (58) |
| 0.000 | 14 | 11.96 | -30 | -85 | 16 | L Middle Occipital Gyrus | hOc4lp (27) |
| 0.000 | 12 | 10.98 | -30 | -34 | 58 | L Postcentral Gyrus | 4p (54) |
| 0.000 | 12 | 10.9 | 27 | -67 | 34 | R Superior Occipital Gyrus |  |
| k<10 | | | | | | | |
| 0.000 | 4 | 11.14 | -21 | -73 | 31 | L Superior Occipital Gyrus |  |
| 0.001 | 3 | 11.27 | -57 | 8 | 31 | L Precentral Gyrus | Area 44 (45) |
| 0.001 | 3 | 10.35 | -30 | -19 | 55 | L Precentral Gyrus |  |
| 0.000 | 5 | 10.18 | -39 | -19 | 55 | L Precentral Gyrus | 4a (27) |
| 0.001 | 3 | 9.73 | -36 | -73 | -8 | L Inferior Occipital Gyrus | FG1 (26) |

***Action observation***

In the AO condition, activations (*Table 3sm*) – in terms of 0^th^ order effects – were seen in the inferior, middle and superior occipital gyri, middle temporal gyrus, inferior and superior parietal lobules, and postcentral gyri. Activations in the precentral gyri, insula and cerebellar areas, for example, did not survive corrections for multiple comparisons. FWE-corrected effects for higher orders were found in the postcentral gyrus, supramarginal gyrus, insula, middle temporal gyrus and inferior frontal gyrus (Broca area) for the -1^st^ order; inferior parietal lobule and caudate for the +3^rd^ order; and inferior parietal lobule, middle temporal gyrus, precentral cortex, and occipital and fusiform cortices for the -3^rd^ order. No significant effects were found for the +1^st^, +2^nd^, +4^th^, -2^nd^ and -4^th^ orders at a corrected level.

**Table 3sm| Brain regions that are activated for action observation.** This shows the main effect of hand gripping (0^th^ order) and force-related effects (1^st^, 2^nd^, 3^rd^ and 4^th^ orders) on BOLD signal reported for the five polynomial coefficients that resulted in significant clusters. Regions are corrected p_u_<0.001 (k≥10) at the voxel level. *Clusters in italic have a minor extension (k<10)*. Regions (*) are corrected (p<0.05) for multiple comparisons at the cluster level. The last column reports the probability (%) of these voxels being located in the respective Brodmann area (BA) or specific location (Loc) according to the cytoarchitectonic maps. ext = number of voxels in a cluster; T = T-value at the voxel level. x, y, z are peak coordinates in MNI space (mm).

| **Cluster** | | **Peak** | | | | | |
| --- | --- | --- | --- | --- | --- | --- | --- |
| **p_u_** | **ext** | **T** | **x** | **y** | **z** | **Anatomical region** | **BA/Loc (%)** |
| **0^th^ order** | | | | | | | |
| k≥10 | | | | | | | |
| 0.000 | 382 | 8.64 | 51 | -67 | -2 | R Middle Temporal Gyrus * | hOc4la (51) |
|  |  | 7.8 | 24 | -91 | -5 | R Inferior Occipital Gyrus * | hOc3v [V3v] (24) |
|  |  | 6.23 | 24 | -91 | 10 | R Superior Occipital Gyrus * | hOc3d [V3d] (14) |
| 0.000 | 257 | 8.51 | -21 | -91 | -11 | L Inferior Occipital Gyrus * | hOc3v [V3v] (71) |
|  |  | 8.23 | -45 | -73 | 1 | L Middle Occipital Gyrus * | hOc5 [V5/MT] (54) |
|  |  | 6.83 | -42 | -79 | -5 | L Inferior Occipital Gyrus * | hOc4la (69) |
| 0.024 | 28 | 7.16 | 24 | -85 | 37 | R Superior Occipital Gyrus | hOc4d [V3A] (25) |
| 0.001 | 75 | 6.6 | -30 | -49 | 55 | L Inferior Parietal Lobule * | 7PC (SPL) (31) |
|  |  | 5.88 | -36 | -49 | 64 | L Superior Parietal Lobule * | 7PC (SPL) (70) |
| 0.000 | 119 | 6.3 | 30 | -49 | 58 | R Superior Parietal Lobule * | 7PC (SPL) (41) |
|  |  | 6.08 | 33 | -31 | 43 | R Postcentral Gyrus * | 3a (49) |
| 0.002 | 64 | 6.12 | -54 | -22 | 28 | L Postcentral Gyrus * | PFt (IPL) (47) |
| 0.018 | 31 | 5.58 | -36 | -49 | -14 | L Fusiform Gyrus | FG4 (33) |
| 0.038 | 23 | 5.32 | 54 | 2 | 49 | R Precentral Gyrus |  |
|  |  | 4.58 | 57 | 11 | 40 | R Precentral Gyrus |  |
| 0.107 | 13 | 4.7 | 57 | -16 | 37 | R Postcentral Gyrus | 1 (35) |
| 0.062 | 18 | 4.58 | -24 | -73 | 34 | L Superior Occipital Gyrus |  |
| k<10 | | | | | | | |
| *0.227* | *7* | *5.28* | *-45* | *-40* | *25* | *L Supramarginal Gyrus* | *PFcm (IPL) (25)* |
| *0.198* | *8* | *4.76* | *-12* | *-73* | *-20* | *L Cerebellum* | *Lobule VI (Hem) (100)* |
| *0.198* | *8* | *4.74* | *24* | *-67* | *40* | *R Superior Occipital Gyrus* |  |
| *0.174* | *9* | *4.61* | *-57* | *5* | *31* | *L Precentral Gyrus* | *44 (10)* |
| *0.306* | *5* | *4.6* | *18* | *-73* | *-29* | *R Cerebellum Crus I* | *VIIa crusI (Hem) (78)* |
| *0.227* | *7* | *4.58* | *48* | *-25* | *31* | *R Inferior Parietal Lobule* | *PFt (IPL) (44)* |
| *0.361* | *4* | *4.5* | *12* | *-73* | *17* | *R Calcarine Gyrus* | *hOc3d [V3d] (30)* |
| *0.306* | *5* | *4.46* | *-36* | *-4* | *16* | *L Insula* |  |
| *0.227* | *7* | *4.45* | *-27* | *-85* | *28* | *L Middle Occipital Gyrus* | *hOc4d [V3A] (8)* |
|  |  | *4.26* | *-24* | *-79* | *22* | *L Middle Occipital Gyrus* |  |
| *0.306* | *5* | *4.41* | *-60* | *-46* | *37* | *L Inferior Parietal Lobule* | *PFm (IPL) (51)* |
| **-1^st^ order** | | | | | | | |
| k≥10 | | | | | | | |
| 0.014 | 30 | 5.83 | -57 | -19 | 22 | L Postcentral Gyrus | OP1 [SII] (33) |
|  |  | 4.9 | -60 | -13 | 31 | L Postcentral Gyrus | 3b (28) |
| k<10 | | | | | | | |
| *0.173* | *8* | *7.8* | *-48* | *-43* | *28* | *L Supramarginal Gyrus* | *PFcm (IPL) (24)* |
| *0.277* | *5* | *5.31* | *30* | *23* | *-14* | *R Insula Lobe* | *Fo3 (16)* |
| *0.235* | *6* | *5.11* | *48* | *-43* | *10* | *R Middle Temporal Gyrus* |  |
| *0.173* | *8* | *4.81* | *51* | *26* | *4* | *R Inferior Frontal Gyrus*  *(p. Triangularis)* | *45 (Broca) (37)* |
| **-2^nd^ order** | | | | | | | |
| k<10 | | | | | | | |
| *0.28* | *5* | *5.05* | *24* | *8* | *46* | *R Middle Frontal Gyrus* |  |
| **3^rd^ order** | | | | | | | |
| k≥10 | | | | | | | |
| 0.049 | 19 | 5.02 | -51 | -58 | 46 | L Inferior Parietal Lobule | PGa (IPL) (51) |
| 0.213 | 10 | 5.02 | 9 | 20 | 1 | R Caudate Nucleus |  |
| **-3^rd^ order** | | | | | | | |
| k≥10 | | | | | | | |
| 0.006 | 43 | 8.47 | -48 | -25 | 40 | L Inferior Parietal Lobule | 2 (61) |
|  |  | 7.44 | -39 | -28 | 37 | L Inferior Parietal Lobule | 3a (35) |
| 0.008 | 39 | 5.36 | -15 | -76 | 31 | L Cuneus |  |
|  |  | 4.8 | -27 | -67 | 25 | L Lateral Occipital Cortex |  |
| 0.017 | 30 | 5.25 | -45 | -70 | 7 | L Middle Temporal Gyrus | hOc4la (16) |
| 0.109 | 12 | 5.08 | -27 | -79 | -11 | L Fusiform Gyrus | hOc4v [V4(v)] (72) |
| k<10 | | | | | | | |
| *0.213* | *7* | *5.07* | *-30* | *-13* | *55* | *L Precentral Gyrus* |  |
| *0.213* | *7* | *4.8* | *-24* | *-94* | *7* | *L Middle Occipital Gyrus* | *hOc4lp (43)* |
| *0.247* | *6* | *4.3* | *27* | *-70* | *31* | *R Superior Occipital Gyrus* |  |

***Conjunction***

Tables on conjunction results for main effects with a cluster extension k<10 are reported in **Table 4sm and 5sm**. Detailed tables on conjunction results are reported for linear and non-linear effects (**Table 6sm and 7sm**).

**Table 4sm| AEON: main effects.** The table reports the action execution-observation network (AEON) regions that presented a main effect (0^th^ order). The statistical threshold is set using p<0.05 (k<10) at the cluster level. The last column reports the probability (expressed as a percentage) of these voxels being located in the respective Brodmann area (BA) or specific location (Loc) according to the cytoarchitectonic maps. ext=extension (number of voxels in a cluster); T=T-value at the voxel level. x, y, z are peak coordinates in MNI space (mm).

| **Cluster** | **Peak** | | | | | |
| --- | --- | --- | --- | --- | --- | --- |
| **ext** | **T** | **x** | **y** | **z** | **Anatomical region** | **BA/Loc (%)** |
| **AEON - 0^th^ order** | | | | | | |
| *2* | *3.31* | *-48* | *-37* | *28* | *L Supramarginal Gyrus* | *PFcm (IPL) (11)* |
| *7* | *2.6* | *42* | *-1* | *13* | *R Rolandic operculum* | *OP3 [VS] (13)* |

**Table 5sm| AEON: force-related BOLD effects.** The table reports the action execution-observation network (AEON) regions that presented a force-related effect (FRE). The statistical threshold is set using p<0.05 (k<10) at the cluster level. Regions (*) survived a p<0.05 correction for multiple comparisons at the peak level. The last column reports the probability (expressed as a percentage) of these voxels being located in the respective Brodmann area (BA) or specific location (Loc) according to the cytoarchitectonic maps. ext=number of voxels in a cluster; T=T-value at the voxel level. x, y, z are peak coordinates in MNI space (mm).

| **Cluster** | **Peak** | | | | | |
| --- | --- | --- | --- | --- | --- | --- |
| **ext** | **T** | **x** | **y** | **z** | **Anatomical region** | **BA/Loc (%)** |
| **AEON - FRE** | | | | | | |
| *8* | *9.23* | *-63* | *-46* | *16* | *L Superior Temporal Gyrus ** | *PF (IPL) (17)* |
| *5* | *7.8* | *21* | *-64* | *-50* | *R Cerebellum ** | *Lobule VIIIa (Hem) (55)* |
| *5* | *7.55* | *18* | *14* | *16* | *R Caudate Nucleus ** |  |
| *5* | *7.54* | *-24* | *-22* | *19* | *Thal ** | *Thal: Parietal (1)* |
| *8* | *6.75* | *36* | *-19* | *10* | *R Insular Lobe* | *Area Ig2 (34)* |
| *7* | *5.29* | *15* | *65* | *25* | *R Superior Frontal Gyrus* | *Fp1 (31)* |
| *9* | *5* | *6* | *-55* | *-11* | *Cerebellar Vermis* | *Lobule V (Hem) (93)* |
| *6* | *4.89* | *-12* | *23* | *43* | *L Superior Frontal Gyrus* |  |
| *6* | *4.24* | *-12* | *-31* | *-5* | *L Lingual Gyrus* | *Thal: Temporal (2)* |

**Table 6sm| Brain regions that are activated for both action execution and action observation considering the linear force related effect (Conjunction Linear).** The statistical threshold is set using p<0.05 (k≥10) at the cluster level. The last column reports the probability (%) of these voxels being located in the respective Brodmann area (BA) or specific location (Loc) according to the cytoarchitectonic maps. ext = number of voxels in a cluster; T = T-value at the voxel level. x, y, z are peak coordinates in MNI space (mm).

| **Cluster** | **Peak** | | | | | | |
| --- | --- | --- | --- | --- | --- | --- | --- |
| **ext** | **T** | **x** | **y** | **z** | **Anatomical region** | **BA/Loc (%)** | |
| **Conjunction Linear** | | | | | | | |
| k≥10 | | | | | | |  |
| 10 | 5.97 | -42 | -22 | 61 | L Precentral Gyrus | 4a (16) | |
|  | 5.63 | -42 | -28 | 61 | L Postcentral Gyrus | 1 (42) / 4a (38) | |
| k<10 | | | | | | |  |
| *2* | *7.68* | *-6* | *-10* | *55* | *L Posterior Medial Frontal* |  | |
| *2* | *5.25* | *-27* | *-22* | *70* | *L Precentral Gyrus* |  | |

**Table 7sm| Brain regions that are activated for both action execution and action observation considering the non-linear force related effect (Conjunction Non-Linear).** The statistical threshold is set using p<0.05 (k≥10) at the cluster level. *Clusters in italic have a minor extension (k<10)*. Regions (*) are corrected (p<0.05) for multiple comparisons at the peak level. The last column reports the probability (%) of these voxels being located in the respective Brodmann area (BA) or specific location (Loc) according to the cytoarchitectonic maps. ext = number of voxels in a cluster; T = T-value at the voxel level. x, y, z are peak coordinates in MNI space (mm).

| **Cluster** | **Peak** | | | | | | | | |
| --- | --- | --- | --- | --- | --- | --- | --- | --- | --- |
| **ext** | **T** | **x** | **y** | **z** | | **Anatomical region** | | **BA/Loc (%)** | |
| **Conjunction Non-Linear** | | | | | | | | | |
| k≥10 | | | | | | | | |  |
| 10 | 21.74 | -63 | -46 | 28 | L Supramarginal Gyrus * | | PFm (IPL) (46) | | |
| 26 | 20.07 | 33 | -61 | 40 | R Angular Gyrus * | |  | | |
| 117 | 18.59 | 48 | -22 | 52 | R Postcentral Gyrus * | | 1 (54) | | |
|  | 16.95 | 45 | -28 | 55 | R Postcentral Gyrus * | | 1 (45) | | |
| 188 | 17.84 | -42 | -28 | 64 | L Postcentral Gyrus * | | 1 (55) | | |
|  | 16.71 | -45 | -25 | 61 | L Postcentral Gyrus * | | 1 (70) | | |
|  | 10.74 | -48 | -13 | 52 | L Postcentral Gyrus * | | 1 (13) | | |
|  | 9.19 | -45 | -34 | 52 | L Postcentral Gyrus | | 2 (60) | | |
|  | 8.09 | -48 | -19 | 40 | L Postcentral Gyrus | | 3b (70) | | |
|  | 7.65 | -48 | -22 | 52 | L Postcentral Gyrus | | 1 (26) | | |
|  | 6.53 | -45 | -19 | 34 | L Postcentral Gyrus | | 3b (41) | | |
|  | 5.51 | -36 | -46 | 52 | L Inferior Parietal Lobule | | hIP3 (IPS) (40) | | |
| 50 | 16.67 | -21 | -88 | 19 | L Middle Occipital Gyrus * | | hOc4d [V3A] (40) | | |
| 116 | 16.61 | -48 | -76 | 4 | L Middle Occipital Gyrus * | | hOc4la (79) | | |
| 12 | 3.75 | 33 | -46 | 46 | R Superior Parietal Lobule | | 7PC (SPL) (12) | | |
| 117 | 12.38 | 42 | -55 | -5 | R Inferior Temporal Gyrus * | |  | | |
|  | 5.73 | 48 | -79 | 4 | R Middle Occipital Gyrus | | hOc4la (80) | | |
|  | 5.4 | 45 | -58 | 7 | R Middle Temporal Gyrus | |  | | |
|  | 4.77 | 42 | -64 | 10 | R Middle Temporal Gyrus | |  | | |
|  | 4.4 | 42 | -73 | 10 | R Middle Occipital Gyrus | | hOc4la (51) | | |
| 24 | 7.83 | -9 | -25 | -5 | Thal | | Thal: Premotor (4) | | |
|  | 6.86 | -15 | -25 | -5 | Thal | | Thal: Parietal (88) | | |
| 27 | 4.73 | 36 | 38 | 22 | R Middle Frontal Gyrus | |  | | |
| 24 | 7.65 | -36 | -1 | 1 | L Insular Lobe | |  | | |
|  | 5.29 | -33 | 2 | 10 | L Insular Lobe | |  | | |
|  | 3.92 | -33 | 11 | 7 | L Insular Lobe | |  | | |
| 14 | 7.58 | -18 | -46 | -26 | L Cerebellum (IV-V) | | Lobule V (Hem) (42) | | |
|  | 6.79 | -21 | -49 | -23 | L Cerebellum (IV-V) | | Lobule VI (Hem) (84) | | |
|  | 5.4 | -21 | -58 | -23 | L Cerebellum (VI) | | Lobule VI (Hem) (95) | | |
| 14 | 7.36 | 57 | 5 | 40 | R Precentral Gyrus | | 44 (9) | | |
| 16 | 6.76 | -21 | -82 | 37 | L Superior Occipital Gyrus | | hOc4d [V3A] (34) | | |
|  | 5.7 | -24 | -85 | 34 | L Superior Occipital Gyrus | | hOc4d [V3A] (21) | | |
| 12 | 6.48 | 30 | -40 | 52 | R Inferior Parietal Lobule | | 2 (80) | | |
| 17 | 6.36 | 6 | -16 | 61 | R Posterior-Medial Frontal | |  | | |
|  | 3.34 | 6 | -25 | 55 | R Posterior-Medial Frontal | | 4a (66) | | |
| 15 | 5.83 | 12 | -76 | 52 | R Superior Parietal Lobule | | 7P (SPL) (71) | | |
| 11 | 4.54 | -36 | 35 | 19 | L Middle Frontal Gyrus | | 46 | | |
| k<10 | | | | | | | | |  |
| *9* | *7.29* | *-36* | *-76* | *-26* | *L Cerebellum (Crus 1)* | | *Lobule VIIa crusI (Hem) (100)* | | |
| *9* | *4.89* | *-29* | *-55* | *-14* | *L Fusiform Gyrus* | | *FG3 (70)* | | |
| *5* | *4.8* | *24* | *11* | *67* | *R Superior Frontal Gyrus* | |  | | |
| *7* | *4.8* | *-24* | *-70* | *-29* | *L Cerebellum (Crus I)* | | *Lobule VIIa crusI (Hem) (68)* | | |
| *5* | *4.49* | *-9* | *-4* | *43* | *L Middle Cingulate Cortex* | |  | | |
|  | *4.21* | *-12* | *2* | *40* | *L Middle Cingulate Cortex* | |  | | |
| *5* | *4.34* | *-42* | *-7* | *16* | *L Rolandic Operculum* | | *OP3 [VS] (3)* | | |
|  | *3.5* | *-36* | *-7* | *16* | *L Rolandic Operculum* | | *OP3 [VS] (1)* | | |
| *9* | *4.32* | *45* | *8* | *31* | *R Precentral Gyrus* | | *45 (12)* | | |
| *6* | *4.22* | *18* | *-13* | *76* | *R Superior Frontal Gyrus* | |  | | |
| *5* | *9.43* | *15* | *-25* | *43* | *R Middle Cingulate Cortex* | | *5Ci (SPL) (4)* | | |
| *8* | *9.38* | *-45* | *-40* | *31* | *L Supramarginal Gyrus* | | *hIP2 (IPS) (14)* | | |
| *8* | *3.88* | *-6* | *8* | *58* | *L Posterior-Medial Frontal* | |  | | |
